# Phenoscaping Reveals Multimodal γδ T-cell Cytotoxicity as a Strategy to Overcome Cancer Cell–Mediated Immunomodulation

**DOI:** 10.1158/0008-5472.CAN-25-1890

**Published:** 2025-08-29

**Authors:** Callum Baird Nattress, Rhianna O’Sullivan, Daniel Fowler, Colin Hutton, Petra Vlckova, Jahangir Sufi, Magdalena Buschhaus, Ewa Basiarz, Maria Ramos Zapatero, Ferran Cardoso Rodriguez, Xiao Qin, Ashley Campbell, Angeliki Kanouta, Vivian S.W. Li, Kerry Chester, John Anderson, Marta Barisa, Jonathan P.H. Fisher, Christopher J. Tape

**Affiliations:** 1 Cell Communication Laboratory, Department of Oncology, University College London Cancer Institute, London, United Kingdom.; 2 Innate Immune Engineering Laboratory, Department of Developmental Biology and Cancer, Zayed Centre for Research into Rare Diseases in Children, University College London Ormond Street Institute of Child Health, London, United Kingdom.; 3 Stem Cell and Cancer Biology Laboratory, The Francis Crick Institute, London, United Kingdom.; 4 Antibody Medicines Laboratory, Department of Oncology, University College London Cancer Institute, London, United Kingdom.; 5 Department of Developmental Biology and Cancer, Zayed Centre for Research into Rare Diseases in Children, University College London Great Ormond Street Institute of Child Health, London, United Kingdom.

## Abstract

γδ T cells can kill cancer cells via antibody-independent cytotoxicity (AIC) and antibody-dependent cellular cytotoxicity (ADCC). A better understanding of how these cytotoxic mechanisms are affected by different cancer cells and different T-cell donors could help identify improved immunotherapeutic strategies. To test the combinatorial interactions among T cell interdonor heterogeneity, cancer cell intertumor heterogeneity (ITH), and multimodal γδ T-cell killing, we performed a systematic single-cell phenoscaping analysis of more than 1,000 γδ T-cell and colorectal cancer patient-derived organoid cultures. Single-cell analysis of posttranslational modification (PTM) signaling, cell cycle, apoptosis, and T-cell immunophenotypes revealed that whereas unmodified γδ T cells have limited antitumor activity, IL15Rα–IL15 fusion protein [stabilized IL15 (stIL15)]-engineered γδ T cells can kill patient-derived organoids via AIC without exogenous cytokine support. However, when stIL15 γδ T cells only killed via AIC, cancer cells reciprocally rewired γδ T-cell PTM signal networks in an ITH-specific manner to suppress anticancer cytotoxicity. stIL15 γδ T cells could overcome this cancer cell immunomodulation by also engaging B7-H3–targeted ADCC independent of B7-H3 checkpoint activity. Combined AIC and ADCC rescued γδ T-cell PTM signaling flux and enabled γδ T cells to kill chemorefactory revival colon cancer stem cells. Together, these results demonstrate that multimodal γδ T-cell cytoxicity mechanisms can overcome ITH-specific immunomodulation to kill chemorefractory cancer cells.

**Significance:**

Single-cell phenoscaping of more than 1,000 γδ T-cell and patient-derived organoid cultures shows that cancer cells suppress anticancer γδ T-cell cytotoxicity but γδ T cells can use multimodal killing to overcome immunomodulation.

## Introduction

Adoptive T-cell therapies are an important new class of anticancer immunotherapies (1). Despite the success of αβ chimeric antigen receptor T (CAR-T) cells for the treatment of hematologic malignancies, the efficacy of cellular therapies against solid tumors remains limited (2). Furthermore, the reliance of CAR-T cells on a single killing modality can limit their cytotoxic potential against antigen-heterogeneous cancers (3). Alternative adoptive cellular therapies based on γδ T cells and NK cells are undergoing evaluation as anticancer biotherapeutics, but our understanding of how these cells are regulated by genetic engineering and interactions with heterogenous cancer remains limited.

Vγ9Vδ2 γδ T cells (hereafter γδ T cells) are a subset of cytotoxic T cells that can kill cancer cells via multiple mechanisms (4). Antibody-independent cytotoxicity (AIC) is achieved by both MHC-independent function of the γδ T-cell receptor (TCR) and NK-like receptors such as NKG2D, DNAM-1, and the natural cytotoxicity receptors NKp30, NKp44, and NKp46 (5). Additionally, γδ T cells can perform antibody-dependent cellular cytotoxicity (ADCC) via Fcγ receptor CD16 (FcγRIII) and tumor-bound IgG (6–8). γδ T cells can be genetically engineered (9, 8), are safe in the allogeneic setting (10), and can be readily expanded for clinical use (4). The various γδ T-cell killing modalities, as well as their ability to persist within and serially kill solid tumors, can be further enhanced via engineered secretion of common γ-chain cytokines (9, 8).

Colorectal cancer is a solid tumor of the colonic and rectal epithelium that results in the death of >900,000 people per year (11). Colorectal cancer tumors are complex heterocellular systems that possess substantial nongenetic plasticity (12) and an immunosuppressive tumor microenvironment (TME; ref. 13). Although ∼15% of patients display microsatellite instability (MSI) leading to high tumor mutational burden (TMB) and remarkable neoadjuvant immune-checkpoint blockade (ICB) responses (14), the majority of patients with colorectal cancer have microsatellite stable (MSS) disease with a low TMB and respond poorly to immunotherapies (15). Furthermore, colorectal cancer cells possess high phenotypic plasticity (16, 17) that can drive nongenetic resistance to standard-of-care chemotherapies via stem cell transdetermination from chemosensitive proliferative colonic stem cells (proCSC) to chemorefractory revival CSCs (revCSC; refs. 12, 18). Consequently, there is a desperate need to develop new treatment options for chemoresistant revCSC-dominant MSS colorectal cancer.

A primary challenge in the treatment of cancer is the large variance in phenotype and treatment response between patients—commonly known as “intertumor heterogeneity” (ITH; ref. 19). In the context of allogeneic cellular therapies, the complexity of ITH is further compounded by variability between cell products derived from different peripheral blood mononuclear cell (PBMC) donors—so-called “interdonor heterogeneity” (IDH; refs. 20, 21). How cancer ITH interacts with γδ T-cell IDH across a range of T-cell killing mechanisms is unknown.

Phenoscaping is an emerging experimental technique that combines systematic high-throughput perturbations with high-dimensional single-cell analysis to map the phenotypic regulators of a dynamic cellular system (22). We recently used phenoscaping to map the cell-intrinsic and -extrinsic regulators of CSC plasticity (23), but to date, no phenoscaping study of T-cell–cancer cell interactions has been reported.

In this study, we present a large-scale single-cell phenoscaping analysis of γδ T-cell interactions with patient-derived organoid (PDO) models of colorectal cancer to chart the regulators of multimodal γδ T-cell cytotoxicity across cancer ITH and T-cell IDH. Using thiol-reactive organoid barcoding *in situ* mass cytometry (TOB*is* MC; refs. 24, 25), we studied the posttranslational modification (PTM) signaling, cell state, apoptosis, and immunophenotype of γδ T-cell–PDO interactions in 3D across >1,000 perturbations at single-cell resolution.

Phenoscaping analysis revealed that unmodified γδ T cells have variable expansion phenotypes and poor cytotoxicity against colorectal cancer PDOs. However, γδ T cells engineered to express an IL15Rα–IL15 fusion protein (stabilized IL15, stIL15; refs. 8, 26) are cytotoxic against colorectal cancer PDOs but can be reciprocally immunomodulated by PDOs in an ITH-specific manner, including global rewiring of γδ T-cell PTM signaling networks. We find that tumor-derived γδ T-cell immunomodulation can limit anticancer killing when γδ T cells use AIC alone but that immunomodulation can be overcome by cancer cell opsonization using anti–B7-H3 mAb therapy, which engages γδ T-cell ADCC capacity. Crucially, we find that engineered γδ T cells can kill MSS PDOs that are enriched for chemorefractory revCSCs. Taken together, these results demonstrate the value of high-throughput cellular therapy phenoscaping and reveal that γδ T-cell multimodal cytotoxicity can overcome immunomodulation to kill chemoresistant cancer cells.

## Materials and Methods

### Organoid culture

Colorectal cancer PDOs were obtained from the Human Cancer Models Initiative with written informed consent (Sanger Institute; ref. 27) and expanded in 3 × 25 μL droplets of Growth Factor Reduced Matrigel (Corning, 354230) per well of a 12-well plate (Helena Biosciences, 92412T). Each well was supplemented with 1 mL of PDO expansion media comprising Advanced DMEM F/12 (Thermo Fisher Scientific, 12634010) containing 2 mmol/L L-glutamine (Thermo Fisher Scientific, 25030081), 1 mmol/L N-acetyl-L-cysteine (Sigma, A9165), 10 mmol/L HEPES (Sigma, H3375), 500 nmol/L A83-01 (Generon, 04–0014), 10 mmol/L SB202190 (Avantor, CAYM10010399-10), 1X B-27 Supplement (Thermo Fisher Scientific, 17504044), 1X N-2 Supplement (Thermo Fisher Scientific, 17502048), 50 ng mL^−1^ EGF (Thermo Fisher Scientific, PMG8041), 10 nmol/L gastrin I (Sigma, SCP0152), 10 mmol/L nicotinamide (Sigma, N0636), 1X HyClone penicillin–streptomycin solution (Thermo Fisher Scientific, SV30010), and conditioned media produced as described in ref. 28 at 5% CO_2_, 37°C. L-cells for conditioned media production were obtained from Shintaro Sato (Research Institute of Microbial Diseases, Osaka University; ref. 29). PDOs were dissociated into single cells with 1X TrypLE Express Enzyme (Gibco, 12604013; incubated at 37°C for 20 minutes) and passaged every 5 to 10 days. For the first 24 hours after dissociation, media were also supplemented with 10 μmol/L Rho-associated protein kinase inhibitor (ROCKi; Y-27632, Sigma, Y0503). L-cells for conditioned media production were obtained from Shintaro Sato (Research Institute of Microbial Diseases, Osaka University). To aid cell type–specific visualization and gating, colorectal cancer PDOs were transfected with H2B-RFP (Addgene, 26001).

### Generation of CRISPR B7-H3^KO^ PDOs

A custom lentivirus containing a GFP reporter, Cas9, and guide RNA against B7-H3 (CTG​GTG​CAC​AGC​TTT​GCT​G) was ordered from Merck. PDOs were dissociated into single cells with 1X TrypLE Express Enzyme (Gibco, 12604013; incubated at 37°C for 20 minutes). Single-cell PDOs were transduced at an multiplicity of infection of 5 in PDO expansion media supplemented with 10 μmol/L ROCKi and 8 μg/mL polybrene (Merck, TR-1003-G) with spinoculation at 300 x *g* for 1 hour at room temperature. Following transduction, single-cell PDOs were passaged and expanded as normal. Upon reaching a sufficient number for cell sorting, PDOs were dissociated into single cells and washed in FACS buffer [PBS (Gibco) supplemented with 1 mmol/L (Sigma, 03690), 25 mmol/L HEPES, and 1% FBS]. Cells were stained with 1 μg/mL of anti-human B7-H3 primary antibody (Prof. Kerry Chester, UCL Cancer Institute, London, United Kingdom) and 1 μg/mL of goat anti-human IgG secondary antibody (Thermo Fisher Scientific, A-21445). Cells negative for B7-H3 were bulk sorted through a BD FACSAria III into collection media (50% PDO expansion media and 50% FBS supplemented with 10 μmol/L ROCKi) and grown as normal. PDOs underwent a repeat bulk sort following multiple passages to select a stable B7-H3^KO^ population. 100% B7-H3^KO^ efficiency of the twice-sorted PDO population was confirmed with B7-H3 gene amplification and Sanger sequencing.

### PBMC isolation

This study was approved by a national Ethics Committee (West Midlands HRA, 14/WM/1253) and complied with the World Medical Association Declaration of Helsinki with written informed consent. Leukopaks from healthy donors were purchased from Cambridge Bioscience, and PBMCs were isolated by density-adjusted centrifugation using Lymphopure density gradient medium (BioLegend, 426201). Residual red blood cells were removed using ammonium–chloride–pottasium (ACK) lysing buffer (Thermo Fisher Scientific, A1049201), and platelets were removed with slow-speed centrifugation. PBMCs were immediately frozen in a freezing solution containing 10% DMSO, with storage complying with the Human Tissue Act 2004. Owing to the size and longitudinal nature of the screen, cryopreserved PBMCs were used so that a consistent bank of γδ T cell donors could be utilized across multiple batches of experiments.

### γδ T-cell expansion

Cryopreserved PBMCs were thawed and rested overnight in RPMI 1640 medium supplemented with 1X GlutaMAX and 10% (v/v) FBS (Gibco, 10082147) at 5% CO_2_, 37°C. For Vγ9Vδ2 expansion, overnight-rested PBMCs were plated in tissue culture–treated plates at 2 × 10^6^ cells/cm^2^ in fresh media and stimulated with 5 μmol/L zoledronic acid (Actavis) and IL2 (100 IU/mL; aldesleukin, Novartis). Unless otherwise stated, IL2 was replenished every 2 to 3 days by removing 50% of the media from the well and replacing with fresh media containing IL2 (200 IU/mL). γδs were expanded until day 12 at which point they were harvested for experimental use and flow cytometry immunophenotyping. By day 12, γδ T cells typically represent the predominant cell type, with remaining populations of NK cells and *αβ* T cells varying by donor.

### Lentiviral vectors

Gene constructs containing both enhanced GFP (eGFP) and stIL15 were designed using SnapGene 2.8.3. Gene blocks were synthezised by Twist Bioscience and cloned into a pCCL.SFFV lentiviral vector using standard restriction enzyme cloning. eGFP–stIL15 lentivirus was produced in-house and was titred on HEK293T cells (CVCL_0063) with the number of titratable U/mL determined by flow cytometry.

### Lentiviral transduction of γδ T cells

Following 48 hours of zoledronic acid and IL2 stimulation, lentivirus was diluted in Opti-MEM (Thermo Fisher Scientific, 31985062) and added directly to the γδ T-cell culture wells at a multiplicity of infection of 4. Spinoculation and transduction enhancers were not used for lentiviral transduction. Transduced γδs were expanded until day 12, at which point, they were harvested for experimental use and flow cytometry immunophenotyping. Transduction efficiency was calculated as the percentage of live Vδ2 expressing GFP.

### Flow cytometry

Flow cytometric analysis was performed using a FACSymphony A5 flow cytometer (BD Biosciences, SCR_022538). Data were acquired using BD FACSDiva (v8.0.1) and analyzed using FlowJo v10.10.0 and Cytobank (Beckman Coulter). Compensation was calculated with the use of OneComp eBeads (Thermo Fisher Scientific, 01-1111-41) stained with single-color antibodies. Colorectal cancer PDO B7-H3 antigen density was estimated using the BD Quantibrite PE Kit (BD Biosciences, 340495) as per the manufacturer’s protocol and a primary anti-human B7-H3 PE antibody (Miltenyi, 130-120-712).

### Anti–B7-H3 mAb

The anti–B7-H3 mAb was obtained from a murine single-chain Fv (scFv) phage-display library constructed as previously described (30). B7-H3–binding scFvs were identified by panning and phage monoclonal ELISA. The scFv was subcloned into a chimeric hIgG1 format using a G1m1, 17 heavy chain allotype and produced as a full antibody by Evitria AG (www.evitria.com) using transient expression in CHO cells and protein A purification. Fc-null versions of the same B7-H3 mAb clones were generated via the incorporation of “LALA” mutations into the Fc domain (L234A/L235A) and were also produced by Evitria.

### Coculture of colorectal cancer PDOs with γδ T cells

PDO-γδ T-cell cocultures were always performed with day 12-expanded γδ T cells. On day 7 of γδ T-cell expansion, PDOs were dissociated into single cells with 1X TrypLE Express Enzyme (Gibco, 12604013; incubated at 37°C for 20 minutes) and expanded in 3 × 25 μL droplets of Growth Factor Reduced Matrigel (Corning, 354230) per well of a 12-well plate (Helena Biosciences, 92412T). Each well was supplemented with 1 mL of Advanced DMEM F/12 (Thermo Fisher Scientific, 12634010) containing 2 mmol/L L-glutamine (Thermo Fisher Scientific, 25030081), 1 mmol/L N-acetyl-L-cysteine (Sigma, A9165), 10 mmol/L HEPES (Sigma, H3375), 1X B-27 Supplement (Thermo Fisher Scientific, 17504044), 1X N-2 Supplement (Thermo Fisher Scientific, 17502048), 50 ng mL^−1^ EGF (Thermo Fisher Scientific, PMG8041), 10 nmol/L gastrin I (Sigma, SCP0152), 10 mmol/L nicotinamide (Sigma, N0636), 500 nmol/L A83-01 (Generon, 04–0014), 10 mmol/L SB202190 (Avantor, CAYM10010399-10), and 1X HyClone penicillin–streptomycin solution (Thermo Fisher Scientific, SV30010) at 5% CO_2_, 37°C for 4 days. For the first 24 hours after dissociation, media were also supplemented with 10 μmol/L ROCKi (Y-27632, Sigma, Y0503). On day 11 of γδ T-cell expansion (D5 of PDO expansion), PDOs were starved in reduced media, consisting of Advanced DMEM F/12 containing only 2 mmol/L L-glutamine, 1 mmol/L N-acetyl-Lcysteine, 10 mmol/L HEPES, 1X B-27 Supplement, 1X N-2 Supplement, 10 mmol/L nicotinamide, and 1X HyClone penicillin–streptomycin solution at 5% CO_2_, 37°C for 24 hours before experimental seeding. On the day of experimental seeding, day 5-expanded PDOs and day 12-expanded γδ T cells were seeded as mono- and cocultures in flat-bottom 96-well plates (Helena Biosciences, 92696T) in 50 μL Matrigel stacks with 300 μL of reduced media for 48 hours. PDOs and γδ T cells were seeded at a density of ∼1.5 × 10^3^ and 3.75 × 10^5^ cells/well, respectively. PDOs are complex, heterocellular 3D structures that comprise tens to hundreds of individual cancer cells per PDO, although significant heterogeneity in size and cellular density exist between patients. Single-cell counts from dissociated PDOs suggest an E:T ratio of ∼5:1 to 1:1 in this study, based on a density of 1.5 × 10^3^ PDOs/well. When specified, cultures were treated with 1 μg/mL of anti-human B7-H3 IgG (Prof. Kerry Chester, UCL Cancer Institute).

After 24 hours of culture, all wells received a 50% media change with fresh reduced media. Experiments were ended after 48 hours of coculture and processed for TOB*is* MC (see the following section).

### TOB*is* MC

PDO monocultures, γδ T-cell monocultures, and PDO–γδ T cell cocultures were analyzed using the TOB*is* MC protocol described in Sufi and colleagues (25). In brief, after 48 hours, PDO-γδ T-cell cultures were incubated with 25 mmol/L (5-Iodo-20-deoxyuridine; ^127^IdU; Standard BioTools, 201127) at 37°C for 30 minutes, and 5 minutes before the end of this incubation, 1X Protease Inhibitor Cocktail (Sigma, P8340) and 1X PhosSTOP (Sigma, 4906845001) were added into the media. Each well was then fixed in 4% PFA/PBS (Thermo Fisher Scientific, J19943K2) for 1 hour at 37°C. PDO–γδ T cells were washed with PBS, dead cells were stained using 0.25 mmol/L ^194^cisplatin (Standard BioTools, 201194), and PDO–γδ T cells were barcoded *in situ* with 126-plex (9-*choose*-4) TOB*is* overnight at 4°C. Unbound barcodes were quenched in 2 mmol/L GSH, and all PDO–γδ T cells were pooled. PDO-γδ T cells were dissociated into single cells using 0.875 mg mL^−1^ dispase II (Thermo Fisher Scientific, 17105041), 0.2 mg mL^−1^ collagenase IV (Thermo Fisher Scientific, 17104019), and 0.2 mg mL^−1^ DNase I (Sigma, DN25) in C-Tubes (Miltenyi, 130-096-334) via gentleMACS Octo Dissociator with Heaters (Miltenyi, 130-096-427, SCR_020271). Single PDO and γδ T cells were washed in cell staining buffer (Standard BioTools, 201068) and stained with extracellular rare-earth metal-conjugated antibodies for 30 minutes at room temperature. PDO–γδ T cells were then permeabilized in 0.1% (v/v) Triton X-100/PBS (Sigma, T8787) and 50% methanol/PBS (Thermo Fisher Scientific, 10675112) and stained with intracellular rare-earth metal-conjugated antibodies for 30 minutes at room temperature. PDO–γδ T cells were then washed in cell staining buffer, and antibodies were cross-linked to cells using 1.6% (v/v) FA/PBS for 10 minutes. PDO-γδ T cells were incubated in 125 nmol/L^191^ Ir/^193^ Ir DNA intercalator (Standard BioTools, 201192A) overnight at 4°C. PDO-γδ T cells were washed, resuspended in cell acquisition solution plus (CAS+; Standard BioTools, 201244) with 2 mmol/L EDTA (Sigma, 03690), and analyzed using a CyTOF XT (Standard BioTools, SCR_02634) at 200 to 400 events s^−1^.

### Chemotherapy treatment of colorectal cancer PDOs

We recently performed a phenoscape of >2,500 drug-treated colorectal cancer PDO and cancer-associated fibroblast cultures (18). We leveraged the cytotoxicity data from PDO monocultures treated with a range of physiologically relevant concentrations of SN-38 (1–100 nmol/L), 5-fluorouracil (0.2–200 μmol/L), and oxaliplatin (2–200 nmol/L) as a benchmark of chemosensitivity. The maximum apoptosis response achieved for each chemotherapy was chosen as a comparison for γδ T-cell cytotoxicity.

### MC data processing

Multiplexed FCS files were debarcoded into individual experimental conditions using the Zunder Lab Single Cell Debarcoder tool (https://github.com/zunderlab/single-cell-debarcoder; ref. 31). Debarcoded FCS files were uploaded to Cytobank and gated for Gaussian parameters and DNA content (^191^Ir/^193^). Epithelial PDO cells were gated as Pan-CK^+^EpCAM^+^; γδs were gated as CD45^+^CD3^+^γδ-TCR^+^. Exported PDO and γδ cells were arcsinh transformed and mean-centered across acquisition batches before downstream analysis. The therapeutic apoptosis achieved by γδ T cells was calculated relative to the baseline apoptosis measured in PDO monoculture controls.

### *In vivo* studies: circulating γδ T-cell analysis

Immune-deficient female 6-week-old NOD.Cg-*Prkdc*^*scid*^*Il2rg*^*tm1Wjl*^/SzJ mice were obtained from the Francis Crick Institute Biomedical Research Facility and housed in individually ventilated cages with a maximum of five mice per cage. Mice were used at ∼9 weeks of age for experiments. All experiments were performed with ethical approval (Animal Welfare Ethical Review Body) under Home Office Project License PPL PP4021677. Mice were treated with an intravenous injection into the tail vein of PBS or 10 × 10^6^ unmodified or stIL15-γδ T cells. After 7 days, mice were prepared for terminal cardiac puncture under general anesthesia. Two hours before cardiac puncture, mice received an intraperitoneal injection of 200 μg ^127^IdU to label cells undergoing S-phase. Following cardiac puncture, red blood cells were removed with ACK lysing buffer (Thermo Fisher Scientific, A1049201), and PBMCs were washed in PBS before fixation with 4% PFA/PBS (Thermo Fisher Scientific, J19943K2) at room temperature for 10 minutes. Fixed PBMCs from each mouse were barcoded with unique TOB*is* barcodes for 2 hours at room temperature. Unbound barcodes were quenched in 2 mmol/L GSH before all PBMCs were pooled into a single tube. Cells were then stained for MC as described above.

### Earth mover’s distance and *x̄*Earth mover’s distance

Earth mover’s distance (EMD) scores were calculated using the Python package scprep. Standard EMD scores were calculated between probability distributions (markers) across experimental conditions using defined controls as references. Signs were applied to EMD scores to denote an increase or decrease in expression. *x̄*EMD represents a coordinate sliced Wasserstein distance (32) and describes the absolute average difference across all measured markers between experimental conditions irrespective of direction. *x̄*EMD was calculated by taking the absolute EMD values (no sign applied) for all markers and averaging them. *x̄*EMD scores were calculated between stIL15-γδ T-cell monoculture controls and those in coculture with colorectal cancer PDOs; this allowed the quantification of the average change in stIL15-γδ T-cell immunophenotype, PTM signaling, and cell state due to immunomodulation by PDOs.

### Density-resampled estimate of mutual information and ∆density-resampled estimate of mutual information

*k*-Nearest neighbors density-resampled estimate of mutual information (*k* NN-DREMI; ref. 33) scores were computed with the Python package *scprep* (34). *k* NN-DREMI scores among 11 PTMs were calculated, which yielded a total of 110 PTM-PTM combinations for each stIL15-γδ T-cell condition. To quantify the rewiring of stIL15-γδ T cell PTM signaling networks, the differences in PTM–PTM combinations across conditions were calculated [∆*k* NN-DREMI (referred to as ∆DREMI)]. These pairwise calculations were computed for all conditions, either comparing stIL15-γδ T-cell monocultures to untreated cocultures or untreated cocultures compared with cocultures with B7-H3 mAb. ∆DREMI scores for each condition were visualized via potential of heat diffusion for affinity-based transition embedding (PHATE).

### PHATE

PHATE is a nonlinear dimensionality reduction method that aims to preserve the local and global structure of datasets (35). A variety of data matrices can drive PHATE embeddings, including EMD scores and ∆DREMI scores, whereby the resulting embedding captures maximal variance across all the dataset dimensions. Conclusions can be made surrounding the relative distance of points within a PHATE embedding, which are linked to underlying similarities or differences in the chosen markers of a MC panel (as captured by EMD calculation).

### Single-cell RNA sequencing of colorectal cancer PDOs

Single-cell RNA sequencing (scRNA-seq) of colorectal cancer PDOs was performed using the SPLiT-seq method described in Ramos Zapatero and colleagues (18). In brief, PDOs were cultured in triplicate as described above for 72 hours, with fresh reduced media replaced every 24 hours. PDOs were harvested and dissociated into single cells using TrypLE (Thermo Fisher Scientific, 12604013) incubated for 10 minutes at 37°C on a heated orbital shaker at 300 rpm. Cells were filtered through a 35-μmol/L filter and resuspended in 1 mL of PBS supplemented with 1.25 μL Protectorase RNase Inhibitor (Merck, 3335402001) and 2.5 μL SUPERase RNase Inhibitor (Thermo Fisher Scientific, AM2694). Cells were fixed, permeabilized, and counted and 5% (v/v) DMSO was added before aliquoting and freezing in a Mr Frosty at −80°C. One complete SPLiT-seq experiment was performed per two PDOs studied (five independent SPLiT-seq runs total). Split-pool barcoding combinatorial indexing was performed as per the SPLiT-seq protocol (36) with minor modifications. Briefly, cells were thawed and reverse-transcribed in the barcode-RT1 plate, followed by two rounds of pooling and ligation in the ligation-L2 and ligation-L3 plates. Cells were then counted and aliquoted into sublibraries of approximately 12,500 cells, lysed, and frozen at −80°C until library preparation.

Following split-pool barcoding, four sublibraries were carried forward for cDNA isolation, amplification, and library generation as described in Ramos Zapatero and colleagues (18). Libraries were then pooled together equally, loaded onto an Illumina Novaseq (200 cycle NovaSeq 6000 S2 Reagent Kit v1.5), and sequenced within the following format: R1:74-i7:06-i5:00-R2:86 to yield a paired-end read structure comprising cDNA transcriptomic information in read 1 and barcoding information in read 2. The data were demultiplexed using the sublibrary i7 indexes, and reads were aligned to the GRCh38 reference genome using the zUMIs package (v2.9.7; ref. 37) with STAR (v2.7.3a), filtering on a whitelist of permitted cell barcodes and merging cells that shared PolyA and Random Hexamer RT1 barcodes from the same RT well plate position with identical L2 and L3 barcodes, including reads originating from exons and introns. Cell barcodes were collapsed based on a two hamming distance of close cell barcodes and unique molecular identifiers (UMI) on a 1 hamming distance of UMI sequence. A cell x gene digital gene expression (DGE) matrix was generated for each library.

### scRNA-seq data preprocessing and analysis

For all sequencing runs, the DGE of each sublibrary was processed using the splitRtools package (https://github.com/TAPE-Lab/splitRtools) to annotate cell barcode well locations and sample identities and perform initial quality control. Downstream analysis was performed in Scanpy (38). Sublibrary DGEs were merged, and low-quality cells were excluded using the following parameters; >32,500 UMIs and <1,000 UMIs, >7,000 genes and <300 genes, >20% mitochondrial transcripts, >0.4 UMI/read ratio, and genes detected in fewer than 25 cells. Neotypic doublets were identified and removed using Scrublet (39) with an expected doublet rate of 3%. Cells were then normalized using count-based normalization with a scaling factor of 10,000 excluding highly expressed genes comprising >5% of total counts per cell. The normalized data were then natural log-transformed for downstream analysis. Data were scaled, principal component analysis was performed over 5,000 variable genes, a neighborhood graph was constructed based on the first 50 principal components as input, and Leiden clustering was performed to generate clusters. Once all scRNA-seq datasets had been filtered as above, the final DGEs were merged, ultimately yielding a dataset of 89,894 high-quality single cells.

Using the merged dataset, PDO-specific proCSC and revCSC signatures were computed by scoring each PDO epithelial cell for literature-derived proCSC and revCSC signatures identified by Opzoomer and colleagues (bioRxiv 2024.02.23.581,433) with the score_genes function in Scanpy using default settings on log-normalized data. Scores were computed using a defined set of genes and comparing their average relative expression against a reference set of genes, as previously described in Satija and colleagues (40). Following gene scoring per cell, the stem cell index was calculated by subtracting the proCSC score from the revCSC score to determine the relative distribution of different stem cell signatures within each PDO, as described in Vazquez and colleagues (41).

### Statistical analyses

All statistical analyses were performed using GraphPad Prism v10.1.1. *P* values of less than 0.05 were considered statistically significant.

## Results

### stIL15 engineering supports γδ T cells in a 3D extracellular matrix

**Figure 1.**
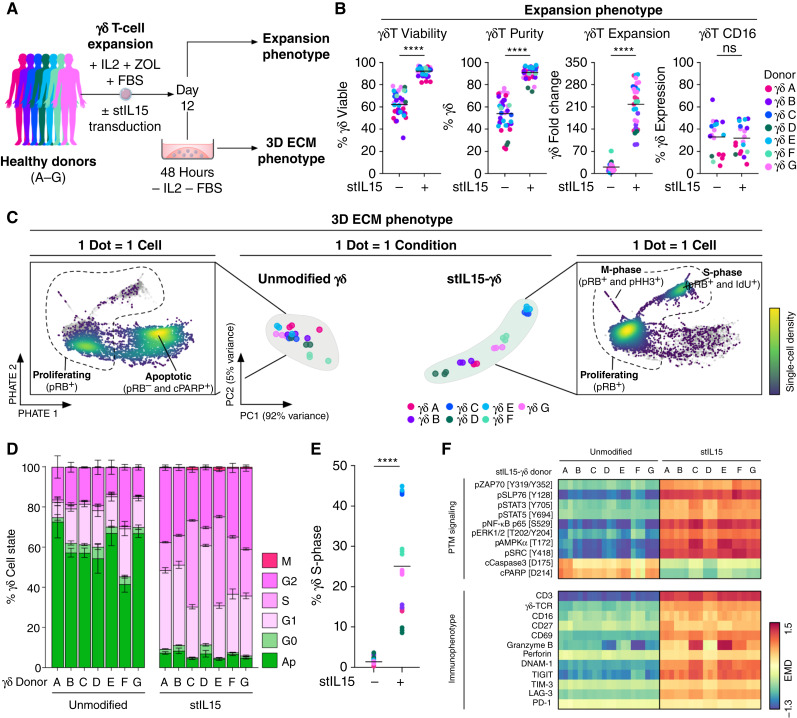
Secreted stIL15 supports γδ T cells without exogenous cytokines in 3D. **A,** Experimental design. γδ T cells were expanded from x7 healthy donors, either unmodified or transduced to overexpress stIL15, cultured in 3D without IL2 or FBS, and analyzed by TOB*is* MC. **B,** Flow cytometry immunophenotyping of unmodified and stIL15-γδ T cells after 12-day expansion (*n* = 38–80 across 7 donors). Comparison by paired *t* test. **C,** Following TOB*is* MC, CD45^+^CD3^+^γδ-TCR^+^ events were gated and exported for analysis. Principal component analysis of x42 3D cultures with single-cell PHATE density embeddings from unmodified and stIL15-transduced γδ T-cell raw expression data. **D,** γδ T-cell states. Mean ± SD of triplicate samples. **E,** γδ T-cell % S-phase. Comparisons by two-way ANOVA with Tukey multiple comparisons test. **F,** EMD heatmap of γδ T-cell PTM signaling and immunophenotyping markers. EMD scores were calculated between unmodified and stIL15-γδ T cells for each donor. ns, not significant; ****, *P* < 0.0001.

Solid cancer TMEs are often nutrient-starved and immunosuppressive (42). In colorectal cancer, the altered metabolic environment can disrupt communication between resident TME leukocytes and contribute toward immunosuppression (43). Therefore, to survive and function within the TME, anticancer cellular therapies must retain viability in a 3D extracellular matrix (ECM) under low-serum conditions. Previous work has demonstrated that γδ T cells engineered to overexpress an IL15Rα–IL15 fusion protein (stIL15) have increased expansion and cytotoxicity compared with unmodified γδ T cells in simple 2D culture systems (8). To investigate whether constitutive stIL15 secretion could support γδ T cells in a 3D ECM culture without exogenous growth factor or cytokine support, we expanded γδ T cells from frozen PBMCs of x7 healthy donors (A-G; Supplementary Tables S1 and S2) in either an unmodified state or following lentiviral transduction to secrete stIL15 (Supplementary Fig. S1A–S1D; refs. 8, 26, 44). We then analyzed γδ T-cell phenotypes immediately after expansion (Supplementary Table S3) and following 48 hours of culture in 3D Matrigel without IL2 or serum support (Fig. 1A).

Immediately following expansion, unmodified γδ T cells were >60% viable, increasing to >90% when transduced with stIL15 (Fig. 1B). stIL15 also increased γδ T-cell purity and expansion but did not alter CD16 expression. Lentiviral transduction efficiencies of >50% were routinely achieved (Supplementary Fig. S1E). These results demonstrate that both unmodified γδ T cells and stIL15-γδ T cells are viable from a range of healthy donors immediately following expansion.

By contrast, we found that unmodified γδ T cells and stIL15-γδ T cells had dramatically divergent phenotypes following serum and cytokine-depleted 3D culture (Fig. 1C). We have previously demonstrated that TOB*is* MC enables high-throughput analysis of PTM signaling, cell state, and apoptosis of 3D heterocellular cultures (24, 25). In this study, we applied TOB*is* MC to quantify the immunophenotype of γδ T cells in 3D ECM (Table S4). TOB*is* MC analysis revealed that unmodified γδ T cells were 40% to 70% apoptotic (cPARP [D214]^+^) after 48 hours in 3D culture without IL2 or serum (Fig. 1D). By comparison, stIL15-γδ T cells were 90% to 95% viable in 3D and retained between 10% to 45% S-phase entry (IdU^+^) following 48-hour culture, compared with <5% in unmodified γδ T cells (Fig. 1E). γδ T-cell S-phase correlated with transduction efficiency, highlighting the donor-specific nature of γδ T cell phenotypes (Supplementary Fig. S1F).

EMD is an optimal transport metric that can quantify shifts between probability distributions of markers across different experimental conditions (45, 46). EMD analysis of unmodified and stIL15-γδ T cells across all donors revealed that stIL15 activated pSTAT3 (Y705)^+^, pSTAT5 (Y694)^+^, pZAP70 (Y319/Y352)^+^, and pSLP76 (Y128)^+^ across all donors. Furthermore, stIL15 upregulated several T-cell activation markers, including granzyme B, perforin, and CD69 across all donors (Fig. 1F). Although engineering did not affect postexpansion %CD16 expression (Fig. 1B), stIL15-γδ T cells maintained higher CD16 expression relative to unmodified controls following serum and cytokine starvation in 3D culture (Fig. 1F). Changes in γδ T-cell immunophenotype and PTM signaling were not induced by viral exposure during transduction and were dependent on stIL15 expression (Supplementary Fig. S1G–S1I).

Similar results were observed *in vivo*. Whereas unmodified γδ T cells rapidly perished to undetectable levels, stIL15-γδ T cells continued to persist and even proliferate in the circulation one week following intravenous infusion into tumor-free NOD.Cg-*Prkdc*^*scid*^*Il2rg*^*tm1Wjl*^/SzJ mice (Supplementary Fig. S2A–S2D). Moreover, evaluation of transduced (GFP^+^) versus nontransduced (NT; GFP^–^) γδ T-cells from stIL15-γδ T-cell infused mice showed that transduced γδ T cells support and maintain the NT γδ T-cell component of the product *in vivo* (Supplementary Fig. S2E–S2G). In addition to enhanced survival of NT γδ T-cell bystanders, stIL15 expression increased GFP^+^ cell NKp30, CD16, CD69, and checkpoint receptor expression (Supplementary Fig. S2H). Collectively, these results demonstrate that stIL15 improves the expansion, viability, and cytotoxic potential of γδ T cells across a range of healthy donors in 3D culture.

### Phenoscaping analysis of γδ T-cell colorectal cancer PDO cytotoxicity

Phenoscaping is an emerging experimental approach that integrates systematic high-throughput perturbations with high-dimensional single-cell analysis to identify phenotypic regulators within dynamic cellular systems (22). Recently, we applied phenoscaping to uncover the cell-intrinsic and -extrinsic regulators of CSC plasticity (23). However, to date, phenoscaping has not been applied to map interactions between T cells and cancer cells.

**Figure 2.**
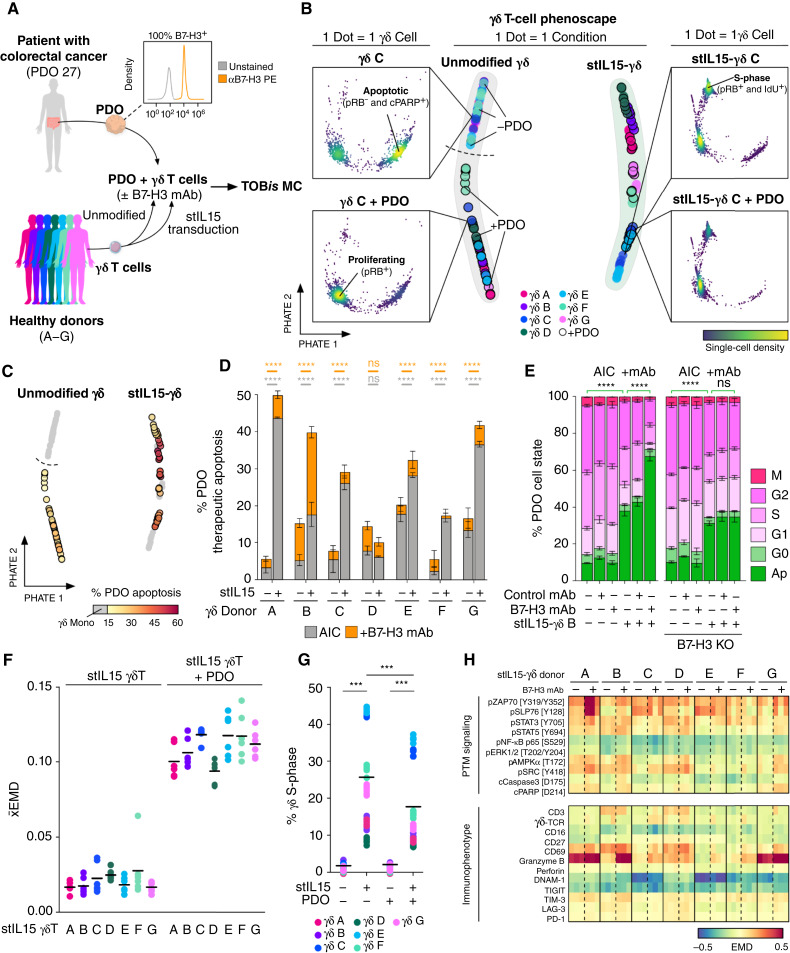
Single-cell phenoscaping of γδ T-cell anti-PDO cytotoxicity. **A,** Experimental design and PDO27 B7-H3 expression measured by flow cytometry. **B,** γδ T-cell EMD PHATE at phenoscape (middle) and single-cell (insets) resolution. ×180 γδ T-cell–PDO cultures. EMD scores were calculated relative to unmodified-γδ monoculture controls to capture changes during stIL15 transduction and γδ coculture with PDO. **C,** γδ T PHATE annotated by PDO apoptosis. **D,** Therapeutic apoptosis via AIC or ADCC per γδ T-cell donor. Mean ± SD of triplicate samples. Two-way ANOVA with Sidak multiple comparisons test. The gray bar compares AIC; the orange bar compares cytotoxicity from AIC + B7-H3 mAb. **E,** ADCC killing in parental and B7-H3 CRISPR knockout PDO 27 demonstrate antigen specificity of ADCC killing. Unpaired *t* test (apoptosis values). Mean ± SD of quintuplet samples. **F,***x̄*EMD of γδ T cells in monoculture or coculture with PDO27. Mean of six replicate samples per donor. The reference for *x̄*EMD scores for all conditions, including monoculture conditions, was calculated relative to monoculture controls. **G,** γδ T-cell S-phase ± PDOs. Six replicates per donor, with overall mean across all donors plotted. Two-way ANOVA with Tukey multiple comparisons test. **H,** EMD heatmap of stIL15-γδ T-cell PTM signaling and immunophenotyping markers ± B7-H3 mAb compared with monoculture controls for each stIL15-γδ T-cell donor. EMD scores were calculated relative to monoculture controls for each donor and treatment. ns, not significant; ***, *P* < 0.001; ****, *P* < 0.0001.

To systematically explore γδ T-cell cytotoxicity, we performed a phenoscaping analysis of γδ T cells from x7 healthy donors (A-G), ± stIL15, ± coculture with an MSI^+^ colorectal cancer PDO 27 (PDO27). To investigate the contribution of ADCC, γδ T cells and PDO27 were also cultured ± 1 μg/mL anti–B7-H3 mAb (Fig. 2A). B7-H3 is a tumor-associated antigen that is overexpressed in multiple solid cancers (47), including colorectal cancer (48), and is being targeted as a tumor-associated antigen in several clinical trials (49). This systematic phenoscaping approach allowed us to study γδ T-cell IDH, stIL15 engineering, AIC, and ADCC killing in a single experiment.

Single-cell analysis of x180 PDO-γδ T-cell 3D cultures revealed that γδ T-cell phenotype is influenced by a range of factors, including PBMC donor, stIL15 transduction, and interactions with PDOs (Fig. 2B). We found that γδ T cells induced PDO apoptosis in a donor-specific manner (Fig. 2C) and stIL15 significantly increased AIC and total anti-PDO cytotoxicity in the presence of B7-H3 mAb in 6/7 donors (Fig. 2D). The therapeutic apoptosis achieved by γδ T cells was calculated relative to the baseline apoptosis measured in PDO monoculture controls. We found that against this MSI^+^ PDO, the majority of stIL15-γδ T-cell killing was performed via AIC, although ADCC could further increase PDO killing in 6/7 donors (Fig. 2D) independent of %CD16 expression in both unmodified and stIL15-γδ T cells (Supplementary Fig. S3A). This suggests that γδ T cells kill colorectal cancer cells via multiple cytotoxic mechanisms (Supplementary Fig. S3B and S3C). Interestingly, stIL15-γδ T cells used a greater proportion of AIC to achieve maximal therapeutic apoptosis relative to unmodified γδ T cells (Supplementary Fig. S3B). CRISPR knockout of PDO B7-H3 inhibited ADCC, demonstrating stIL15-γδ T-cell ADCC is antigen-specific (Fig. 2E). Moreover, AIC against B7-H3-KO colorectal cancer was identical to B7-H3^+^ colorectal cancer, suggesting that—in the context of this assay—B7-H3 does not exert cytotoxicity-suppressing checkpoint effects over γδ T cells.

Unlike small molecules or biologics, cellular therapies are dynamic biological systems that can be regulated by their environment. To quantify how PDOs reciprocally alter γδ T cells, we calculated the absolute mean EMD (*x̄*EMD) for each stIL15-γδ donor when interacting with cancer cells. *x̄*EMD represents a coordinate sliced Wasserstein distance (32) and provides the mean difference between two cell populations for the absolute value of all markers measured—in this case, immunophenotype, PTMs, and cell state, irrespective of direction. Compared with stIL15-γδ T-cell monocultures, *x̄*EMD found that PDOs consistently alter the immunophenotype, PTM expression, and cell state of unmodified and stIL15-γδ T cells across all donors (Fig. 2F; Supplementary Fig. S3D). In unmodified γδ T cells, coculture was predominantly associated with decreased apoptosis and a general increase in PTM signaling (Supplementary Figs. S3E, S4A, and S4B), although decreases in granzyme B and perforin occurred (Supplementary Fig. S4B), which may be due to the poor cytotoxicity by unmodified γδ T cells or immunomodulation (Fig. 2D). Notably, PDOs decreased both apoptosis and S-phase in stIL15-γδ T cells, implying a slowing of cell-cycle progression (Fig. 2G; Supplementary Figs. S3E and S4A). EMD analysis of stIL15-γδ T cells relative to monoculture controls revealed that stIL15-γδ T-cell PTM signaling and immunophenotype are altered by PDOs (Fig. 2H). Most donors experienced increased pZAP70 and pSLP76 signaling during both AIC ± mAb, alongside decreased pNF-κB and pERK signaling as well as decreased expression of both DNAM-1 and TIGIT, although donor-specific changes did occur (Fig. 2H). When assessing the underlying markers of *x̄*EMD scores across all stIL15-γδ T-cell donors, decreases in IdU, DNAM-1, and TIGIT and increases in granzyme B underwent the most immunomodulation (Supplementary Fig. S4C). Collectively, these observations suggest that whereas stIL15-γδ T cells are potent PDO killers, cancer cells exert reciprocal effects on γδ T-cell signaling and cell state in a γδ T-cell donor–dependent manner.

### PDOs reciprocally immunomodulate γδ T-cell phenotypes in a patient-specific manner

**Figure 3.**
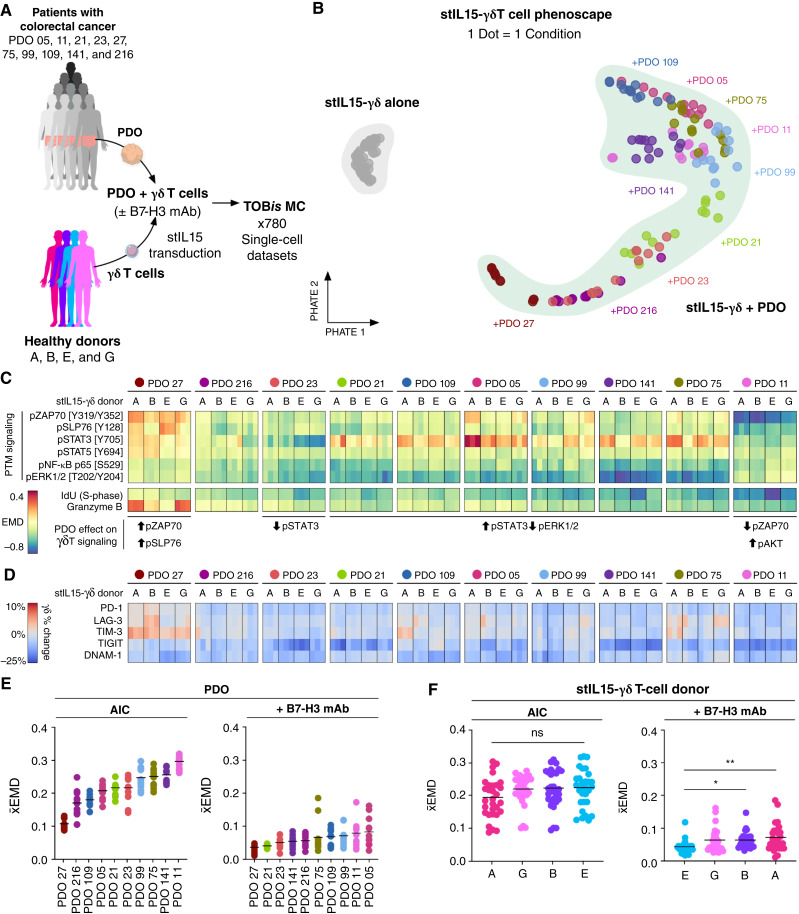
PDOs reciprocally regulate γδ T-cell phenotypes in a patient-specific manner. **A,** Experimental overview. **B,** stIL15-γδ EMD-PHATE phenoscape annotated by PDO. EMD scores were calculated relative to stIL15-γδ monoculture controls to capture changes during γδ coculture with PDO. **C,** γδ T-cell PTM and immunophenotype response to PDOs, including changes in S-phase (IdU). **D,** Percentage change in immunophenotype marker expression in response to PDOs relative to monoculture controls. **E** and **F,***x̄*EMD of γδ T cells killing PDOs either by AIC or in the presence of B7-H3 mAb, colored by PDO (**E**), or γδ T-cell donor (**F**). One-way ANOVA with Tukey multiple comparisons test. *, *P* < 0.05; **, *P* < 0.01; ns, not significant.

To investigate whether the immunomodulation of γδ T cells by PDOs is a generalizable phenomenon of colorectal cancer–γδ T-cell interactions, we performed an additional phenoscaping study of stIL15-γδ T cells from x4 proliferative and cytotoxic donors (A, B, E, and G; Supplementary Fig. S5A; Figs. 1D, E, and 2C and D) ± ×10 different colorectal cancer PDOs, ± anti-B7-H3 mAb (Fig. 3A). By using this systematic approach, we could directly compare the effects of intertumor heterogeneity (ITH), IDH, and multimodal γδ T-cell killing in a single experimental design. Single-cell analysis of >5 million cells across x780 PDO-γδ T-cell cultures revealed that γδ T-cell signaling was determined not by γδ T-cell IDH but rather by ITH-specific interactions with PDOs (Fig. 3B; Supplementary Fig. S5B).

Paired EMD scores were calculated between donor-matched stIL15-γδ T cells in monoculture or coculture with colorectal cancer PDOs, with a positive or negative score indicating the upregulation or downregulation of a marker by PDOs. Generally, PDOs negatively regulated γδ T-cell PTM activatory signaling (pZAP70, pNF-κB, and pERK), effector function (granzyme B), and cell state in a patient-specific manner (Fig. 3C). Interestingly, γδ T-cell pSTAT3 was upregulated by multiple PDOs, consistent with previous reports of elevated STAT3 signaling and pSTAT3 communication between immune cells and colorectal cancer (50, 51). PDOs also induced a global reduction in γδ T-cell S-phase (Fig. 3C) and checkpoint receptor expression (Fig. 3D). The extent of stIL15-γδ modulation during AIC differed based on the PDO, with PDOs 11, 141 and 75 orchestrating the greatest change in γδ T-cell phenotype (Fig. 3E). γδ T cells were consistently altered by PDOs across all γδ T-cell donors, with only minor evidence of IDH, highlighting the universal nature of immunomodulation by colorectal cancer cells (Fig. 3F). This suggests that cancer cell ITH is dominant over γδ T-cell IDH with regards to stIL15-γδ T-cell signaling.

Crucially, despite the widespread downregulation of γδ T-cell signaling by PDOs, the addition of anti-B7-H3 mAb rescued γδ T-cell immunomodulation across all PDOs and PBMC donors (Fig. 3E and F; Supplementary Fig. S5C–S5I). These results suggest that when stIL15-γδ T cells rely solely on AIC, their signaling and cell state can be regulated by PDOs. However, when γδ T cells also engage ADCC, they can overcome cancer cell immunomodulation to restore their cell state and PTM signaling networks.

### Reciprocal inhibition of stIL15-γδ T-cell signaling limits anti–colorectal cancer cytotoxicity

**Figure 4.**
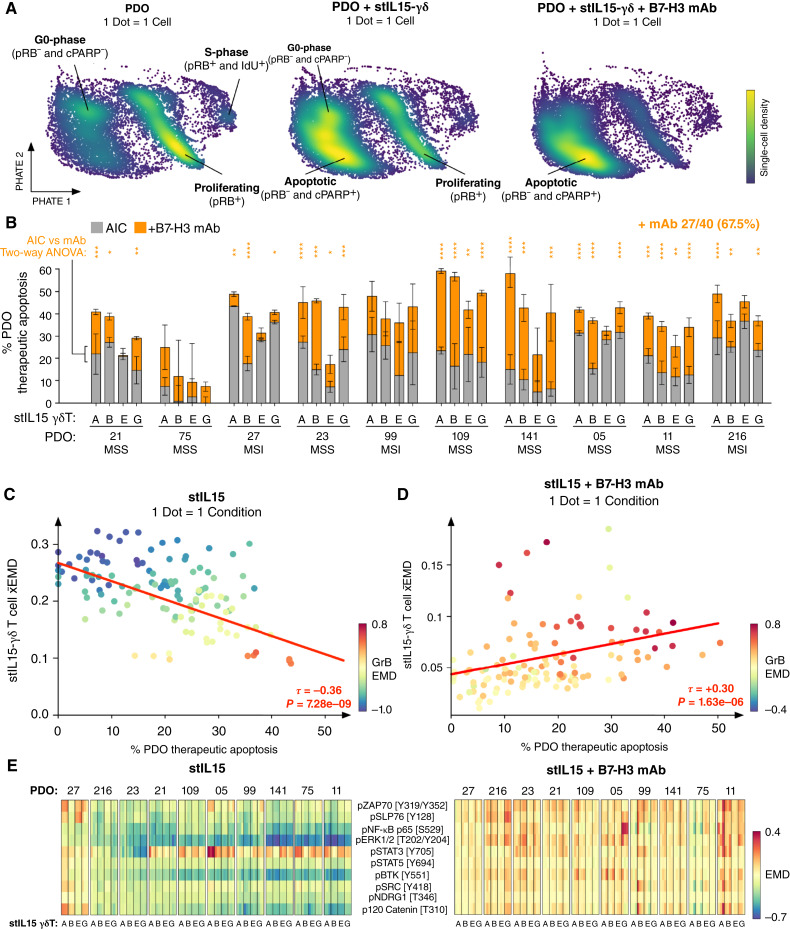
Regulation of γδ T cells correlates with anti-PDO cytotoxicity. **A,** Single-cell PHATE of x10 PDOs + stIL15-γδ T cells ± B7-H3 mAb. **B,** PDO apoptosis via AIC or ADCC per γδ T donor across x10 PDOs. Two-way ANOVA with Sidak multiple comparisons test. Mean ± SD of triplicate samples. **C,** Correlation of γδ *x̄*EMD with anti-PDO cytotoxicity during AIC, colored by γδ granzyme B. τ denotes the Kendall rank correlation coefficient. **D,** Correlation of γδ *x̄*EMD with anti-PDO cytotoxicity + B7-H3 mAb, colored by γδ granzyme B. τ denotes the Kendall rank correlation coefficient. **E,** stIL15-γδ PTM signaling with PDOs ± B7-H3 mAb. *, *P* < 0.05; **, *P* < 0.01; ***, *P* < 0.001; ****, *P* < 0.0001.

The immune synapse between T cells and cancer cells involves multiple protein–protein interactions that govern downstream γδ T-cell intracellular signaling cascades that determine anticancer cytotoxicity. We, therefore, sought to understand whether the immunomodulation of stIL15-γδ T cells by PDOs is associated with changes in anti-PDO cytotoxicity. In monoculture controls, PDOs were largely proliferative (pRB^+^). However, when exposed to stIL15-γδ T-cell AIC, PDO cells entered G0 (pRB^–^) and apoptosis (cPARP^+^). PDO apoptosis is further increased when stIL15-γδ T cells also perform ADCC via anti–B7-H3 mAb (Fig. 4A; Supplementary Fig. S6A).

By phenoscaping γδ T cells across multiple donors and patients, we could observe the respective impact of IDH and ITH on γδ T-cell cytotoxicity. As expected, the susceptibility of PDOs to either AIC or ADCC varied across patients and γδ T-cell donors (Fig. 4B; Supplementary Fig. S6B), with differences in ADCC not explained by PDO B7-H3 expression. Although most PDOs were susceptible to AIC, anti–B7-H3 mAb significantly improved cytotoxicity in 67.5% of PDOs (Fig. 4B). Understanding which patients benefit from additional cytotoxicity in the presence of anti–B7-H3 mAb is therefore of importance.

ADCC is a dynamic process that is partially governed by target antigen positivity, target antigen density, effector FcγR expression, and antibody concentration. Work by ourselves and others has previously demonstrated that γδ T-cell %CD16 expression and the degree of antigen expression strongly correlated with ADCC (8).

The susceptibility of PDOs to stIL15-γδ T-cell killing did not correlate with stIL15-γδ T-cell transduction efficiency, viability, or %γδ T-cell purity at harvest from the manufacture. This suggests that subtle variability in stIL15-γδ phenotypes between manufacturing batches did not account for the differences in killing observed (Supplementary Fig. S7A). All γδ T-cell donors expressed CD16, but specific levels of %CD16 expression did not positively correlate with ADCC across the 10 colorectal cancer PDOs tested using 1 μg/mL of anti–B7-H3 mAb (Supplementary Fig. S7A; ref. 8). There were significant differences in antibody-mediated killing despite the 10 PDOs being 100% positive for extracellular B7-H3 expression (Supplementary Fig. S7B). Although B7-H3 antigen density varied across PDOs (Supplementary Fig. S7C), PDOs with higher B7-H3 antigen density were only susceptible to increased killing by γδ T-cell donors E and G (Supplementary Fig. S7D). %CD16 expression by γδ T cells at the experiment endpoint also did not correlate with ADCC (Supplementary Fig. S7E). Despite being an activation marker with dynamic expression, the change in %CD16 following the addition of mAb (relative to coculture controls) also did not correlate with anti-PDO cytotoxicity (Supplementary Fig. S7F). Together, these findings suggest that the susceptibility of 3D colorectal cancer PDOs to ADCC is not determined solely by B7-H3 antigen density and/or γδ T-cell Fc receptor expression in the presence of 1 μg/mL mAb.

Because of their high TMB, MSI colorectal cancer tumors typically respond well to immune checkpoint inhibition, whereas MSS colorectal cancer tumors with a low TMB typically do not (14). Due to the MHC- and neoantigen-independent function of the γδ-TCR, Vγ9Vδ2 T cells have the potential to indiscriminately kill both MSI and MSS colorectal cancer tumors, rendering them attractive candidates for universal cytotoxicity against all colorectal cancer subsets. We found that stIL15-γδ T cells generally performed more AIC against MSI PDOs than MSS PDOs (Supplementary Fig. S7G and S7H). However, when stIL15-γδ T cells gained additional activation through B7-H3 opsonisation, this dichotomy disappeared, with stIL15-γδ T cells killing both MSI and MSS PDOs equally (Supplementary Fig. S7H). Furthermore, the addition of anti–B7H3 mAb increased cytotoxicity for both MSS and MSI PDOs (Supplementary Fig. S7H).

By phenoscaping γδ T cell PTM signaling and cancer cell death across x4 donors and x10 PDOs, we were able to observe broad trends in γδ T-cell biology that are not specific to a single donor or PDO. Collectively, we found that across all donors and PDOs, anti-PDO AIC decreases when cancer cells suppress γδ T-cell signaling (Fig. 4C). This suggests that reciprocal immunomodulation of stIL15-γδ T cells limits anticancer cytotoxicity when γδ T cells use AIC alone. However, when stIL15-γδ T cells also engage ADCC against PDOs, stIL15-γδ T-cell proinflammatory signaling is restored and anticancer cytotoxicity is increased (Fig. 4D). We found that whereas γδ T cells using AIC alone experienced global downregulation of PTM signaling when encountering PDOs, AIC + ADCC reactivated pZAP70, pSLP76, pNF-kB, pSTAT, pMAPK, and PI3K signaling flux and increased granzyme B expression across all γδ T-cell donors (Fig. 4E; Supplementary Fig. S8A and S8B). These results suggest that the addition of exogenous opsonizing anti-B7-H3 mAb to stIL15-γδ T cells can overcome cancer cell immunomodulation.

### Anti–B7-H3 mAb facilitates ADCC over ICB

**Figure 5.**
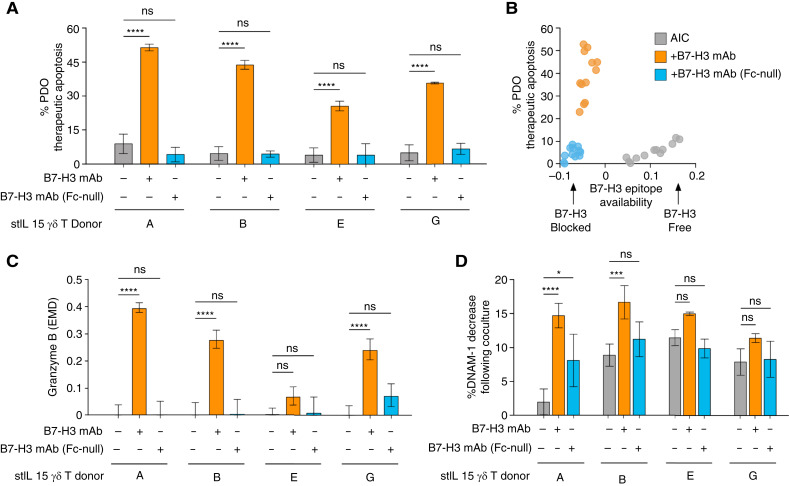
B7-H3 mAb facilitates Fc receptor–dependant ADCC by stIL15-γδ T cells. **A,** Therapeutic apoptosis by stIL15-γδ T cells ± mAb ± mAb (Fc null), against colorectal cancer PDO 141. Two-way ANOVA with Dunnett multiple comparisons test. Mean ± SD of triplicate samples. **B,** Correlation between therapeutic apoptosis and B7-H3 epitope availability. The same clone of B7-H3 mAb was conjugated into the CyTOF staining panel to calculate the relative availability of B7-H3 epitope. **C,** EMD scores of stIL15-γδ T-cell granzyme B expression calculated relative to untreated coculture controls. One-way ANOVA with Sidak multiple comparisons test. Mean ± SD of triplicate samples. **D,** Decrease in stIL15-γδ T-cell % DNAM-1 expression relative to monoculture controls for each treatment. One-way ANOVA with Sidak multiple comparisons test. Mean ± SD of triplicate samples. *, *P* < 0.05; ***, *P* < 0.001; ****, *P* < 0.0001; ns, not significant.

B7-H3 can act as an immune checkpoint during innate and adaptive immune responses in certain contexts (52, 53). To investigate whether B7-H3 mAb cytotoxicity occurs through ADCC and/or blockade of immune checkpoint inhibition, we cocultured stIL15-γδ T cells with colorectal cancer PDOs with anti-B7-H3 IgG1 and an isogenic clone containing a mutated Fc domain that has a significantly reduced affinity for FcγRs on γδ T cells. TOB*is* MC analysis revealed that PDO killing was only achieved in the presence of B7-H3 mAb with functional Fc domains, with Fc-null mAb achieving no significant cytotoxicity over AIC controls (Fig. 5A). Crucially, both IgG1 and Fc-null antibodies blocked the same B7-H3 epitope on colorectal cancer PDOs, indicating equal IgG paratope binding to PDO B7-H3 (Fig. 5B). Furthermore, significant increases in stIL15-γδ T-cell granzyme B expression were only observed in the presence of B7-H3 mAb with functional Fc domains (Fig. 5C), correlating with anticancer ADCC (Fig. 5A). Functional decreases in stIL15-γδ T-cell DNAM-1 expression were also observed upon coculture with PDO. There was a trend toward greater functional regulation of DNAM-1 expression in the presence of B7-H3 mAb with functional Fc domain (Fig. 5D). Alongside B7-H3 antigen CRISPR knockout data (Fig. 2E), these data collectively demonstrate that B7-H3 mAb engages ADCC rather than immune checkpoint inhibition, resulting in reduced immunomodulation of stIL15-γδ T cells by colorectal cancer PDOs (Fig. 4C–E).

### Colorectal cancer PDOs rewire stIL15-γδ T-cell signaling networks

To characterize how colorectal cancer PDO immunomodulation dysregulates stIL15-γδ T-cell PTM signaling networks, we computed DREMI (33, 34) scores for all PTM–PTM pairs in stIL15-γδ T cell ± colorectal cancer PDOs ± B7-H3 mAb. The difference in DREMI score (∆DREMI) for each PTM–PTM pair was calculated between stIL15-γδ T cell cocultures and monoculture controls to assess immunomodulation of signallng networks by colorectal cancer PDOs.

∆DREMI–PHATE analysis revealed that colorectal cancer PDOs rewire γδ T-cell PTM–PTM relationships in a patient-specific manner, with stIL15-γδ T cells from different donors converging on PDO-specific ∆DREMI profiles (Supplementary Fig. S9A and S9B). By contrast, the addition of B7-H3 mAb did not alter PTM–PTM relationships in any γδ T cell donors (Supplementary Fig. S9C–S9E). When combined with PTM signaling activity analysis (Fig. 4C–E), these results suggest that colorectal cancer PDOs induce patient-specific rewiring of stIL15-γδ T-cell PTM signaling networks during AIC immunomodulation, and this is unchanged by anti-B7-H3 ADCC. However, anti–B7-H3 ADCC provides an increased immunostimulatory signaling flux (within altered PTM networks) that permits increased γδ T-cell effector function and degranulation (Supplementary Fig. S9).

### Multimodal stIL15-γδ cytotoxicity kills chemoresistant revival stem cell PDOs

5-Fluorouracil, oxaliplatin, and irinotecan are standard-of-care chemotherapies for colorectal cancer. All chemotherapies target mitotic processes and are therefore more effective in killing proliferative cancer cells compared with slow-cycling cancer cells. It has recently been shown that colorectal cancer tumors possess high levels of nongenetic plasticity (16) and that colorectal cancer cells can rapidly switch between mitotic proCSCs and slow-cycling revCSCs (23). Subsequent work has shown that proCSC are chemosensitive, whereas revCSC are chemorefractory (18). Due to their chemoresistant nature, new therapeutic strategies to kill slow-cycling revCSC are urgently needed (12).

**Figure 6.**
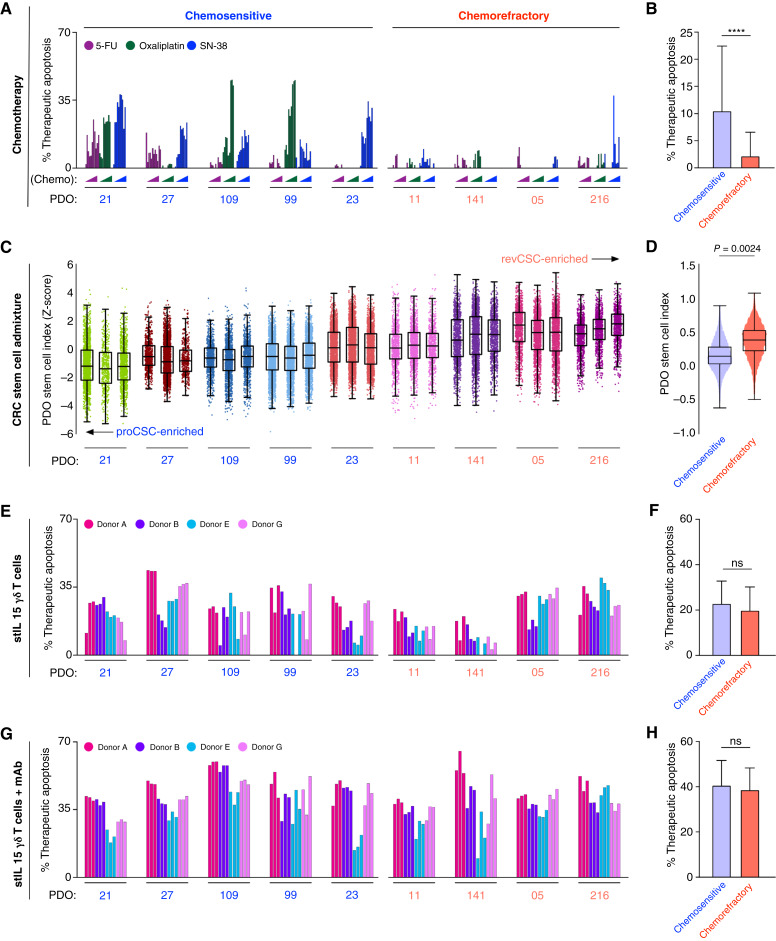
γδ T cells can kill chemorefractory revCSC colorectal cancer PDOs. **A** and **B,** Chemotherapy-induced apoptosis per PDO across a range of chemotherapy concentrations [SN-38, 1–100 nmol/L; 5-fluorouracil (5-FU), 0.2–200 μmol/L; oxaliplatin, 2–200 nmol/L; ref. 18]. **C** and **D,** scRNA-seq stem cell index scores (*Z*-score) of colorectal cancer PDOs ranked from proCSC- to revCSC-enriched. 1 dot = 1 cell. Box and whisker = median + quartiles. Unpaired *t* test between mean PDO stem cell indexes. **E–H,** Therapeutic apoptosis by stIL15-γδ T cells alone (**E** and **F)** or with B7-H3 mAb (**G** and **H**). Mean ± SD. Comparisons by unpaired *t* test. ****, *P* < 0.0001; ns, not significant.

As γδ T cells do not target cancer cell proliferation, we hypothesized that stIL15-γδ T cells could kill chemorefractory revCSC PDOs. To test this, we performed scRNA-seq of the colorectal cancer PDOs used in this study and compared their relative proCSC to revCSC stem cell index (41) to chemotherapy and γδ T-cell killing (Fig. 6). This analysis revealed that colorectal cancer PDOs comprise an admixture of proCSC-like and revCSC-like cells, but PDOs dominated by revCSC cells are more chemoresistant (Fig. 6A–D). However, we found that both stIL15-γδ T AIC (Fig. 6E and F) and ADCC (Fig. 6G and H) can kill colorectal cancer PDOs irrespective of stem cell admixture. γδ T cells that use combined AIC and ADCC multimodal cytotoxicity achieve the largest killing of both proCSC and revCSC colorectal cancer PDOs (Fig. 6G and H). These results suggest that engineered γδ T cells are not susceptible to traditional chemorefractory mechanisms and can kill both chemosensitive proCSC and chemoresistant revCSC cancer cells.

### Multimodal killing protects γδ T cells from reciprocal immunomodulation by cancer cells

Tumor-resident γδ T cells receive a plethora of excitatory and inhibitory signaling cues that modulate their phenotype and anticancer cytotoxicity. Examples include TCR engagement; cytokine and chemokine exposure; Fc receptor engagement; immune checkpoint receptor–ligand engagement; ECM composition; the metabolic environment; and cell–cell interactions. In solid tumors, these cues are orchestrated by cancer cells to maintain an immunosuppressive TME. These cues are complex, combinatorial, and transduced through intracellular T-cell signaling networks to regulate antitumor immune responses. Therefore, as the master regulator of the TME, cancer cells regulate the anticancer cytotoxicity of cellular therapies via the cues that govern their immunophenotype, PTM signaling, and cell state.

**Figure 7.**
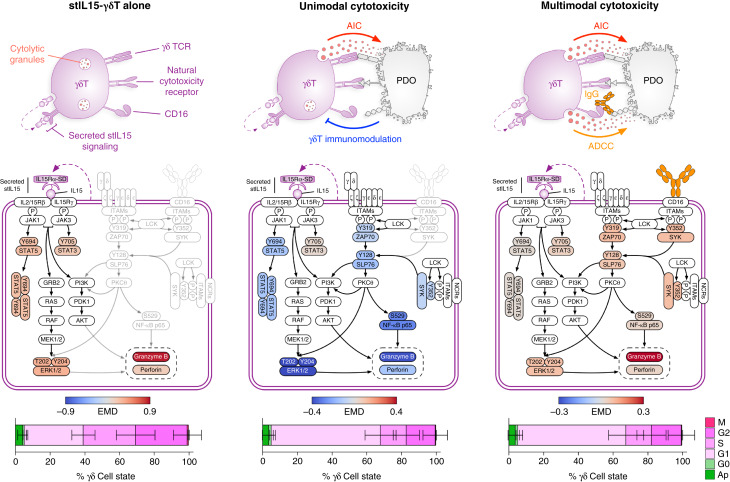
Multimodal γδ T-cell cytotoxicity overcomes cellular therapy immunomodulation. γδ T-cell signaling networks following stIL15 transduction, coculture with PDO, and addition of B7-H3 mAb. Nodes are colored according to γδ EMD scores computed from >4 million cells across 576 conditions. Bottom, γδ cell-state histograms. Each cell-state phase is presented as the mean ± SD (*n* = 129–138). ITAM, immunoreceptor tyrosine-based activation motif.

To understand the overarching signaling dynamics of stIL15-γδ T cells ± colorectal cancer PDOs ± anti–B7-H3 mAb, we compiled data-driven signaling network models from >4 million stIL15-γδ T cells across x576 3D γδ T-cell cultures (Fig. 7). This analysis revealed that in monoculture, stIL15-γδ T cells benefit from constitutive JAK1/3 and STAT3/5 signaling as a result of stIL15 secretion that promotes granzyme B and perforin expression and proliferation (Fig. 7, left). When stIL15-γδ T cells kill PDOs using AIC alone, overwhelming inhibitory cues from cancer cells modulate and suppress γδ T-cell signaling despite continued proliferation (Fig. 7, middle). The specific type of γδ T-cell immunomodulation is regulated by cancer cells, not PMBC donors, highlighting the importance of ITH in regulating T-cell signaling. However, when provided with additional immunoreceptor tyrosine-based activation motif signaling via an anti–B7-H3 mAb/Fc receptor axis, stIL15-γδ T cells can overcome cancer cell signaling repression, activate canonical γδ T-cell PTM flux, and increase cancer cell killing over AIC alone (Fig. 7, right). Collectively, these results suggest that cancer cell γδ T-cell immunomodulation can be overcome using multimodal cytotoxicity.

## Discussion

γδ T cells are gaining traction as an alternative chassis for the development of anticancer biotherapeutics. Their safety in the allogeneic setting, ease of engineering with pseudotyped lentiviral vectors, and variety of anticancer killing modalities provide a complementary alternative to traditional αβ-CAR T cells that rely on unimodal cytotoxicity against antigen-positive disease. Furthermore, γδ T cells have recently been described as immune effectors in MSI colorectal cancer with MHC class I defects following ICB, able to compensate for the lack of conventional CD8^+^ activity in this setting (54). However, despite their initial promise, the treatment of solid tumors with unmodified γδ T cells has failed clinically—warranting the exploration of next-generation engineered γδ T-cell biotherapeutics (55).

Owing to the enormous challenge posed by ITH in cancer, off-the-shelf cellular therapies must be versatile when encountering patient-specific tumors. However, studying the interactions of biotherapeutics with heterogeneous cancer is further compounded by IDH, whereby donor-specific expansion, PTM signaling, and cytotoxicity profiles of cell therapies are determined by the healthy donor they were derived from. Adoptive cellular therapies are complex systems often relying on a plethora of cytotoxicity receptors, with γδ T cells able to kill cancer via multiple modalities, including differential combinations of AIC and ADCC. Understanding the combinatorial interactions between ITH, IDH, and multimodal killing mechanisms is experimentally challenging. In this study, through highly-multiplexed single-cell phenoscaping, we were able to study the interplay between cancer ITH and Vγ9Vδ2 T cell IDH, AIC, and ADCC

TOB*is* MC analysis revealed that unmodified γδ T cells struggle in 3D culture without exogenous cytokine or serum support, with significant apoptosis and poor proliferation after only 48 hours. However, γδ T-cell viability could be rescued via ectopic expression of stIL15. Cytokine engineering of immune cells is becoming a common strategy, with IL15-related technologies being assessed in αβ-CAR T cells (56), NK cells (57), and γδ T cells (8, 58). Superagonism of JAK1/3 and STAT3/5 signaling is thought to promote proliferation, anticancer cytotoxicity, and persistence of biotherapeutics, as well as activation of receptive immune bystanders. Indeed, we show that stIL15 engineering improved γδ T-cell manufacture, with increased proliferation (pRB^+^, IdU^+^), improved T-cell activation (e.g., granzyme B^+^, perforin^+^, and CD69^+^), and decreased apoptosis (cPARP^–^) compared with unmodified γδ T cells (Fig. 1). Moreover, as a secreted product that can benefit receptive immune bystanders, we also found that stIL15 supported and maintained NT γδ T cells *in vivo*, including CD56^+^ NK cells (Supplementary Fig. S2). These results confirm that stIL15 γδ T-cell engineering represents a promising route to overcome several of the limitations of unmodified γδ T cells.

Cancer cell recognition by γδ T cells involves a complex interplay of activatory and inhibitory receptors that is partially governed by the ligands expressed on cancer cells (5). As γδ T cells from different donors also express varying levels of activatory and inhibitory receptors (59), generating universal anticancer γδ T cells from a range of donors is challenging without known biomarkers for donor selection. NK cell IDH has recently been shown to affect clinical outcomes, with biomarkers for donor selection requiring thorough investigation (60). Moreover, unmodified γδ T-cell IDH can contribute toward the varying cytotoxicity of γδ T-cell products both *in vitro* and in human clinical trials (61, 62). Our rationale for stIL15 engineering involves the generation of highly proliferative and universally cytotoxic γδ T cells from a range of donors that can survive in the immunosuppressive TME. Although stIL15 increased S-phase in all γδ T-cell donors, we noticed a range of %S-phase responses that positively correlated with γδ T-cell transduction efficiency (Fig. 1D and E; Supplementary Fig. S1F). This IDH-specific variance could be due to variable RDPro viral binding receptors across different γδ T-cell donors, which are poorly understood in the context of the RDPro envelope.

PDOs are self-organizing heterocellular systems that have gained popularity as a biomimetic 3D model system for the development of biotherapeutics against cancer (63, 64). Unlike simple 2D monolayer cultures, PDOs require candidate biotherapeutics to kill cancer cells in a complex 3D ECM. As it is essential to consider ITH during preclinical development of anticancer therapeutics (19), we chose a panel of colorectal cancer PDOs spanning genetic (e.g., MSI/MSS and *KRAS*/*BRAF* /*APC* status), clinical stage, anatomic location, chemosensitivity, and CSC composition. Interestingly, despite the differences in γδ T-cell IDH, the dominant factor governing γδ T-cell PTM signaling, immunophenotype, and cytotoxicity was cancer cell ITH. These results suggest that cancer ITH is dominant over biotherapeutic IDH. We recently demonstrated that colorectal cancer PDO chemosensitivity aligns with cell-intrinsic cell state and PTM signaling, with slow-cycling PDOs (e.g., PDO 05, 11, 141, and 216) chemorefractory to standard-of-care drugs (18). In this study, we show that chemorefractory PDOs have a higher revCSC-to-proCSC ratio. To our surprise, stIL15-γδ T cells can kill all colorectal cancer PDOs (Fig. 4B)—including chemorefractory revCSC-dominant MSS colorectal cancer, which represents patients with the greatest unmet clinical need (Fig. 6; Supplementary Fig. S7H). To our knowledge, this is the first demonstration that cellular therapies can kill chemorefractory revCSCs. Furthermore, despite orchestrating MHC neoantigen-independent cytotoxicity, stIL15-γδ T cells also performed greater AIC against MSI PDOs (Supplementary Fig. S7H). Collectively, these results reinforce γδ T cells as a promising chassis for engineered adoptive cellular therapies against solid tumors, particularly if cytotoxicity can be performed against slow-cycling chemorefractory cell types.

Studies have investigated the expression of CD16 on γδ T cells against a variety of antigens expressed on both liquid and solid tumors (6–8, 58). Recently, CD16 expression has gained attention as a biomarker of γδ T-cell cytotoxicity even in the absence of mAb, whereby CD16^hi^ donors performed substantially more AIC compared with CD16^lo^ donors in models of ovarian cancer. However, γδ T cells engineered to express higher CD16 had no increase in their cytotoxicity, implying that the natural CD16^hi^ or CD16^lo^ status of γδ T cells is predictive of their cytotoxicity (58). Using 1 μg/mL anti–B7-H3 mAb, we were unable to explain the differential susceptibility of colorectal cancer PDOs to ADCC based on the stIL15-γδ T %CD16 expression either after manufacture or at the experiment endpoint (Supplementary Fig. S7A, S7E, and S7F). Using a B7-H3^KO^ PDO, we demonstrated that ADCC was B7-H3 antigen-specific and that the presence of B7-H3 in the parental PDO did not reduce the AIC performance of stIL15-γδ T cells (Fig. 2E), despite reports that B7-H3 may inhibit the cytotoxicity of γδ T cells via downregulation of IFNγ and granzyme B in colorectal cancer (65). Furthermore, PDOs with higher B7-H3 antigen density were only susceptible to increased killing in the presence of B7-H3 mAb by two γδ T-cell donors (Supplementary Fig. S7C and S7D). Encouragingly, we saw potent ADCC against colorectal cancer PDOs with relatively low B7-H3 density (<20,000 antigens/cell). Previous work assessing anti–B7-H3 CAR T cells for the treatment of B7-H3^+^ pediatric solid and liquid cancers demonstrated cytotoxicity against only medium and high B7-H3 antigen density models (66).

Within the immunosuppressive TME, adoptive cell therapies must navigate a complex interplay of both excitatory and inhibitory environmental signaling cues that govern their proliferation and cytotoxicity. Engineering strategies to “armor” adoptive cell therapies to either augment their function or prevent TME inhibition include the secretion of cytokines, BiTEs, and immune checkpoint inhibitors (67). We provided γδ T cells with both stIL15 and exogenous mAb immunostimulatory agents to promote γδ T-cell proliferation, anticancer cytotoxicity, and resistance to cancer cell suppression (Fig. 7). We found that during AIC, stIL15-γδ T cells were vulnerable to a global reduction in S-phase (Fig. 3C) that was not associated with cell-cycle exit or apoptosis (Supplementary Fig. S4A), demonstrating opposition to the mitogenic effect of stIL15 by colorectal cancer PDO immunomodulation. Surprisingly, colorectal cancer PDOs also exerted a reduction in stIL15-γδ T-cell checkpoint receptor expression (Fig. 3D), which may be due to reduced TCR activation (68) following the immunomodulation that contributed toward poor AIC. However, in the presence of anti–B7-H3 mAb, these reductions in S-phase and checkpoint receptor expression were at least partially overcome (Supplementary Fig. S8), allowing increased cytotoxicity. For these reasons, future work combining B7-H3 mAb with immune checkpoint inhibitors would be particularly interesting. Recently stIL15-γδ T cells were also engineered to secrete an scFv-Fc against GD2 on *in vitro* and *in vivo* models of osteosarcoma, providing an ADCC self-autonomous biotherapeutic (8). Furthermore, the self-secretion of opsonin also engaged ADCC-competent immune bystanders, including NK cells, M1 macrophages, and neutrophils. Efficacy was observed against antigen-heterogeneous disease *in vivo* that was not controlled by a validated CAR T-cell model (8). Aside from more traditional mAb and scFv-Fc proteins, a variety of immune cell engagers are in development to encourage immune cell interaction with cancer, with specific engagers for γδ T cells, NK cells, and myeloid cells being assessed (69). Interestingly, approaches involving the FcγRs, CD16, and CD64; the natural cytotoxicity receptors, NKp30 and NKp46; and NKD2D are undergoing preclinical evaluation, with many already entering clinical trials (69). These alternative engagers have direct compatibility with stIL15-γδ T cells and could be used as additional armoring strategies to provide immunostimulatory signaling cues via tumor-associated antigens.

We demonstrate that opsonising colorectal cancer PDOs with B7-H3 mAb allows stIL15-γδ T cells to overcome cancer cell immunomodulation and perform potent cytotoxicity despite rewiring of PTM signaling networks by colorectal cancer PDOs. Therefore, engineering strategies that promote immunostimulation via the engagement of stabilized CD16 expression and more potent CD16 signaling may be of additional importance when designing ADCC-competent biotherapeutics, especially if they potentiate cytotoxicity in immunomodulating environments with rewiring of PTM signaling by cancer (70, 71). Enzymatic cleavage of CD16 by the matrix metalloproteinase ADAM17 can be prevented by genetic modification of CD16 (72), which has been demonstrated in γδ T cells (58). Furthermore, CD16 polymorphisms with increased affinity for mAb Fc binding (e.g., F158V polymorphism; ref. 73) can be chosen during engineering of noncleavable constructs (74), with a variety of noncleavable, high-affinity CD16-engineered NK cells entering clinical trials (70). Our central finding that using multiple killing mechanisms increases anticancer cytotoxicity and reduces γδ T-cell suppression supports future efforts to engage the polyfunctional nature of γδ T cells in adoptive cell therapies.

Despite the advent of multiple adoptive T-cell therapies engineering approaches, translating promising *in vitro* and *in vivo* findings into the clinic remains a major challenge (75). This complexity is further compounded by cancer ITH and T-cell IDH. A detailed mechanistic understanding of how candidate cell therapies interact with biomimetic 3D models is emerging as an important preclinical step in biotherapeutic development. Through highly-multiplexed single-cell phenoscaping of hundreds of γδ T-cell–cancer cell interactions—assessing PTM signaling, cell state, apoptosis, and immunophenotype—we discovered that stIL15-γδ T cells that only kill by AIC can be immunomodulated by cancer cells in a ITH-dependant manner. However, stIL15-γδ T cells can overcome cancer cell immunomodulation by engaging ADCC to rapidly kill chemorefractory colorectal cancer cells. These results demonstrate the power of systematic preclinical phenoscaping of cellular therapies and reveal that multimodal γδ T-cell killing can overcome cancer cell immunomodulation.

## Supplementary Material

Figure S1 γδT cell stIL15 transduction phenotypes

Figure S2 Phenotypes of unmodified and stIL15-γδ T cells in vivo

Figure S3 Phenotype of stIL15-γδ T cells when co-cultured with CRC PDO27

Figure S4 stIL15-γδs phenotype regulation by CRC PDOs with or without anti-B7-H3 mAb

Figure S5 Regulation of stIL15-γδs signalling and immunophenotype by CRC PDOs with or without anti-B7-H3 mAb

Figure S6 CRC PDO cell-state and γδT cell cytotoxic modality

Figure S7 Relationship between stIL15-γδ cytotoxicity and γδ T cell and PDO phenotypes

Figure S8 stIL15-γδ T cell regulation by CRC PDOs with or without anti-B7-H3 mAb

Figure S9 DREMI rewiring of stIL15-γδ T cell signalling networks by CRC PDOs across all stIL15-γδ T cell donors

Table S1 PBC donor metadata

Table S2 PBMC donor phenotype

Table S3 Flow cytometry reagents for γδT cell phenotyping

Table S4 TOBis MC antibodies

## Data Availability

Raw and processed CyTOF data and illustrations are available as a Community Cytobank project 1551 (https://community.cytobank.org/cytobank/projects/1551). The raw scRNA-seq data generated in this study are publicly available at BioProject PRJNA1308308 (https://www.ncbi.nlm.nih.gov/bioproject/1308308). Processed scRNA-seq data generated in this study are publicly available at Zenodo project 15827759 (https://zenodo.org/records/15827759). All other raw data generated in this study are available upon request from the corresponding author. Code to reproduce figures in this article can be found at https://github.com/callumnattress/Nattress-et-al-Phenoscaping.
