# Supplementary figures and images for: Phenoscaping Reveals Multimodal γδ T-cell Cytotoxicity as a Strategy to Overcome Cancer Cell–Mediated Immunomodulation
Source: Cancer Res. 2025 Aug 29;85(22):4415–32. doi: 10.1158/0008-5472.CAN-25-1890 (PMC12616246; doi:10.1158/0008-5472.CAN-25-1890)

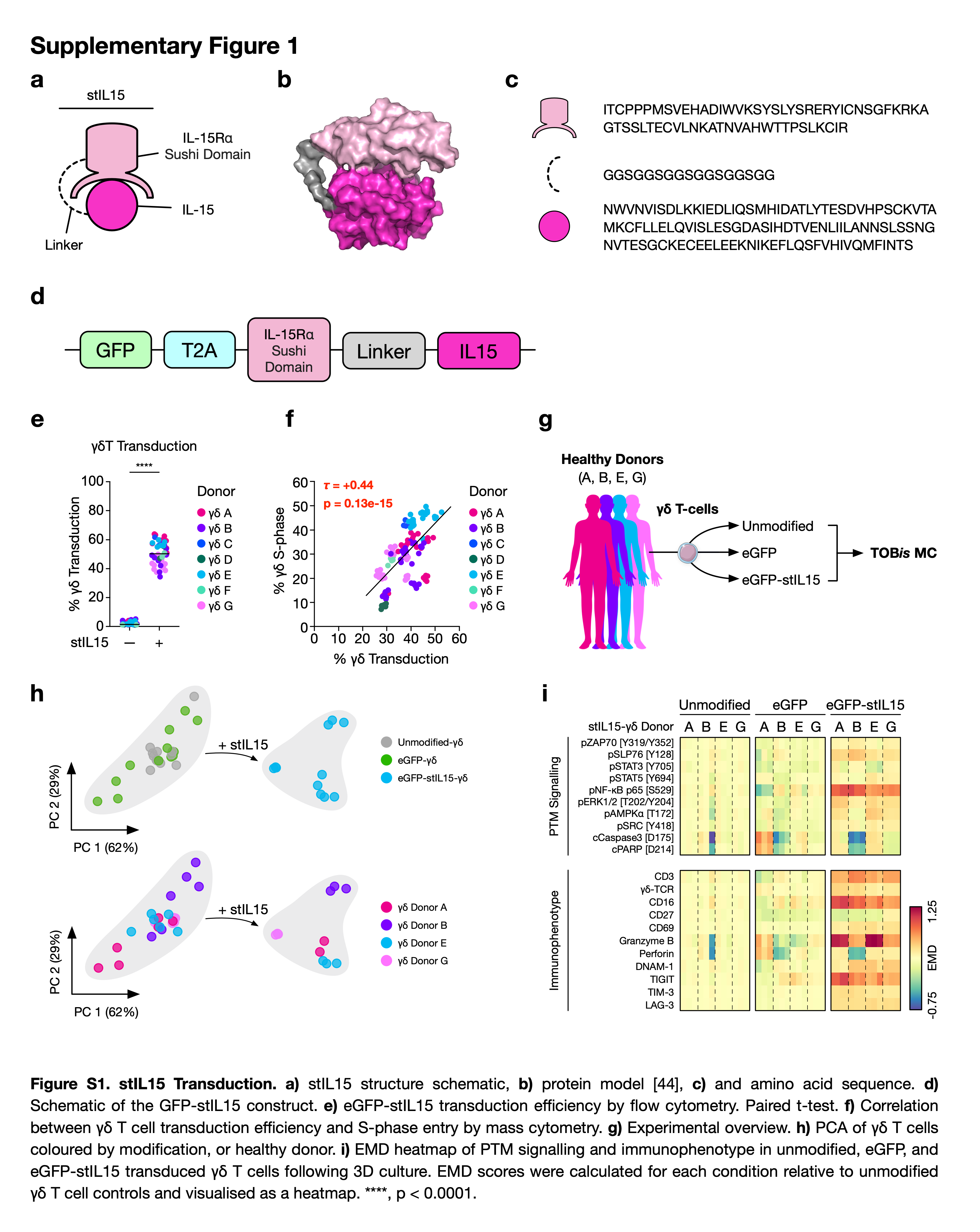

Supplement: Figure S1 — γδT cell stIL15 transduction phenotypes [file can-25-1890_figure_s1_suppsf1.png]

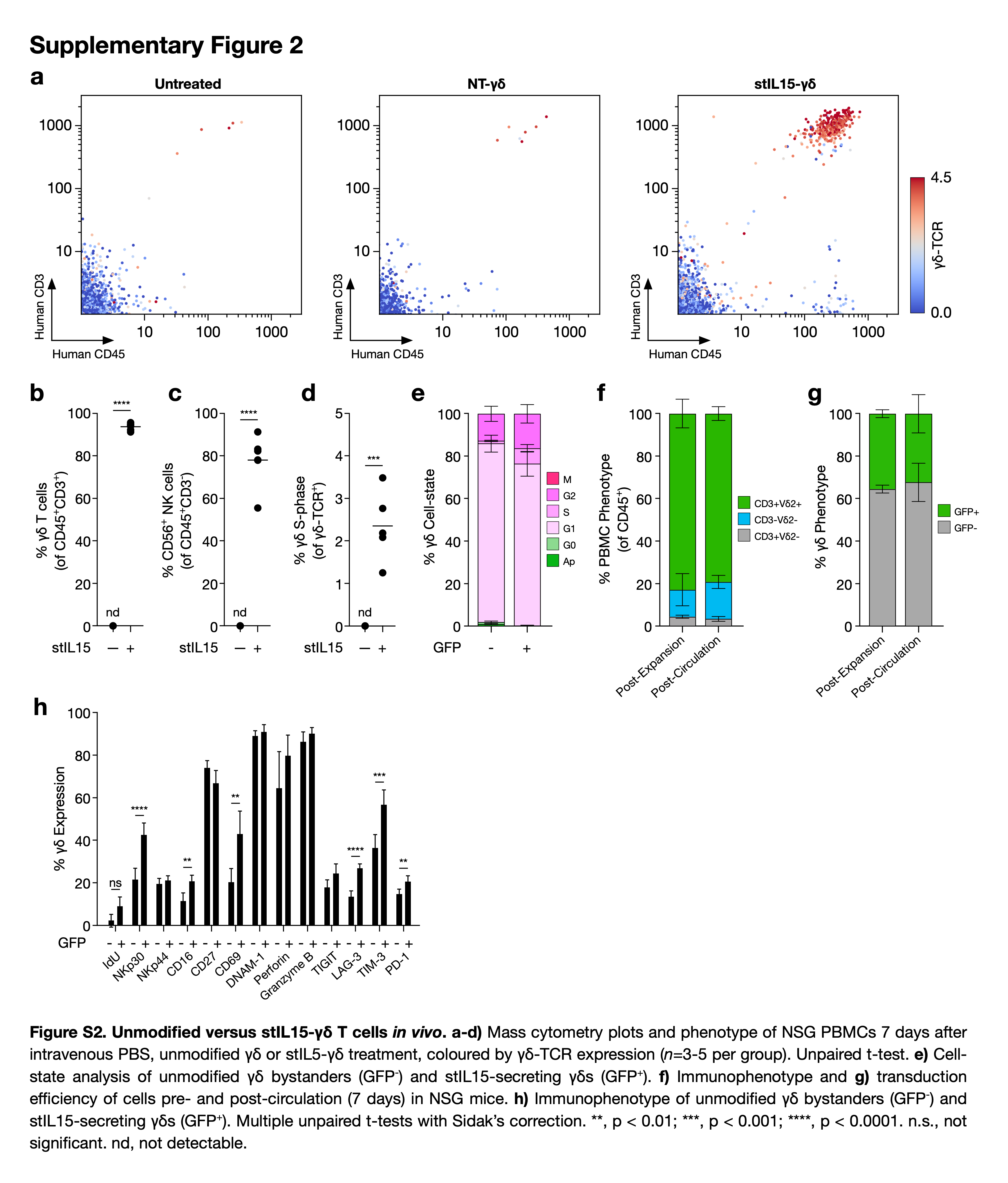

Supplement: Figure S2 — Phenotypes of unmodified and stIL15-γδ T cells in vivo [file can-25-1890_figure_s2_suppsf2.png]

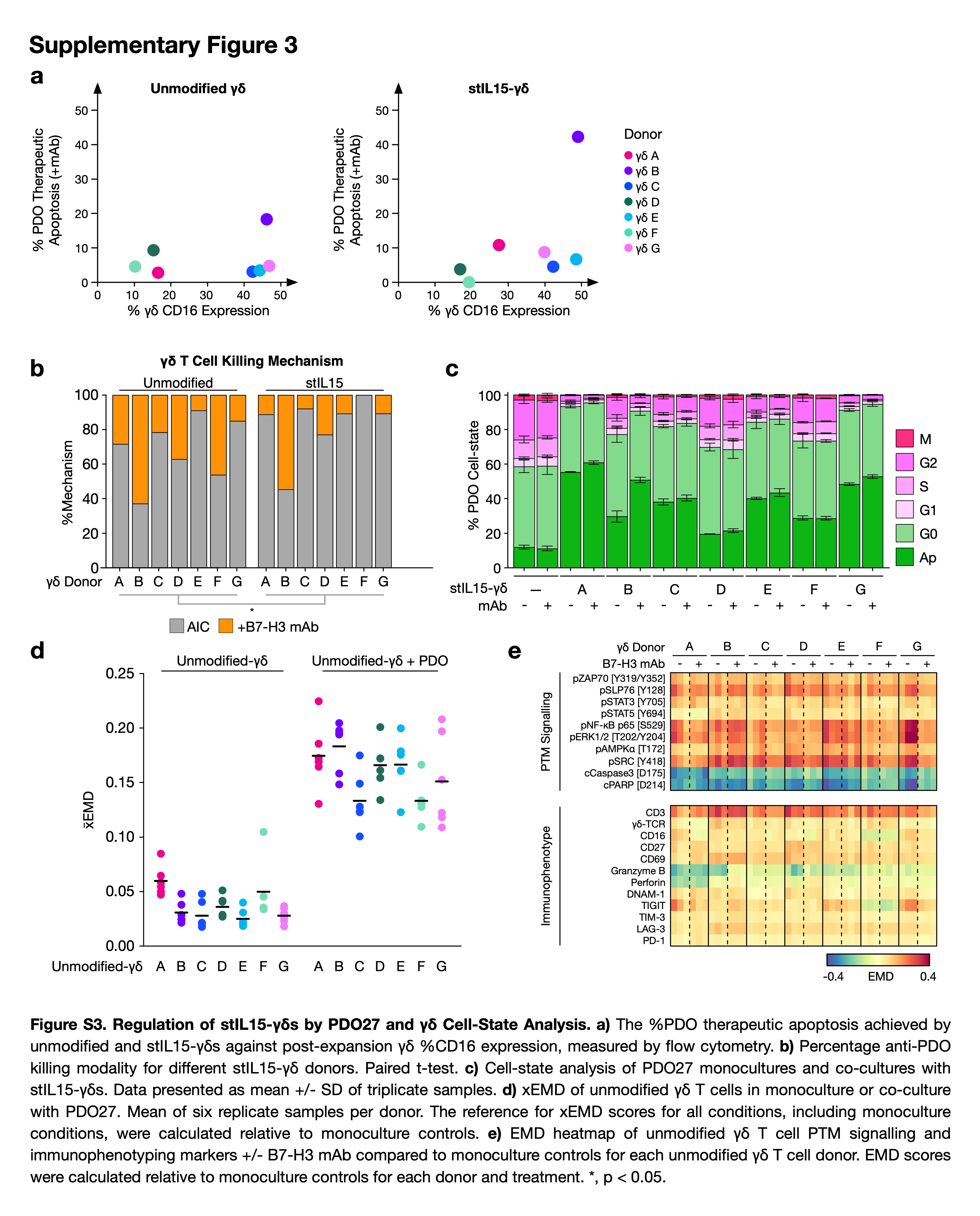

Supplement: Figure S3 — Phenotype of stIL15-γδ T cells when co-cultured with CRC PDO27 [file can-25-1890_figure_s3_suppsf3.png]

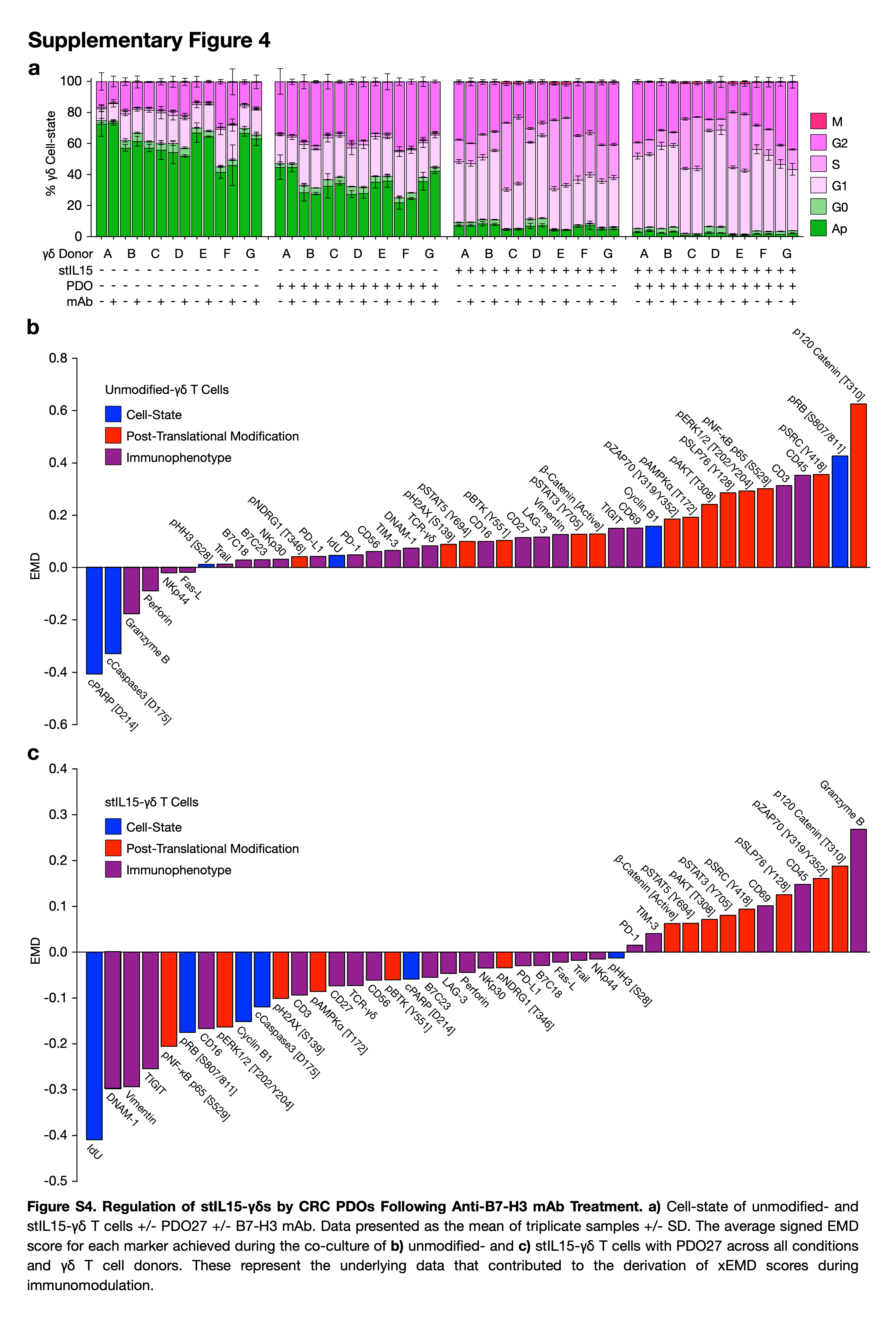

Supplement: Figure S4 — stIL15-γδs phenotype regulation by CRC PDOs with or without anti-B7-H3 mAb [file can-25-1890_figure_s4_suppsf4.png]

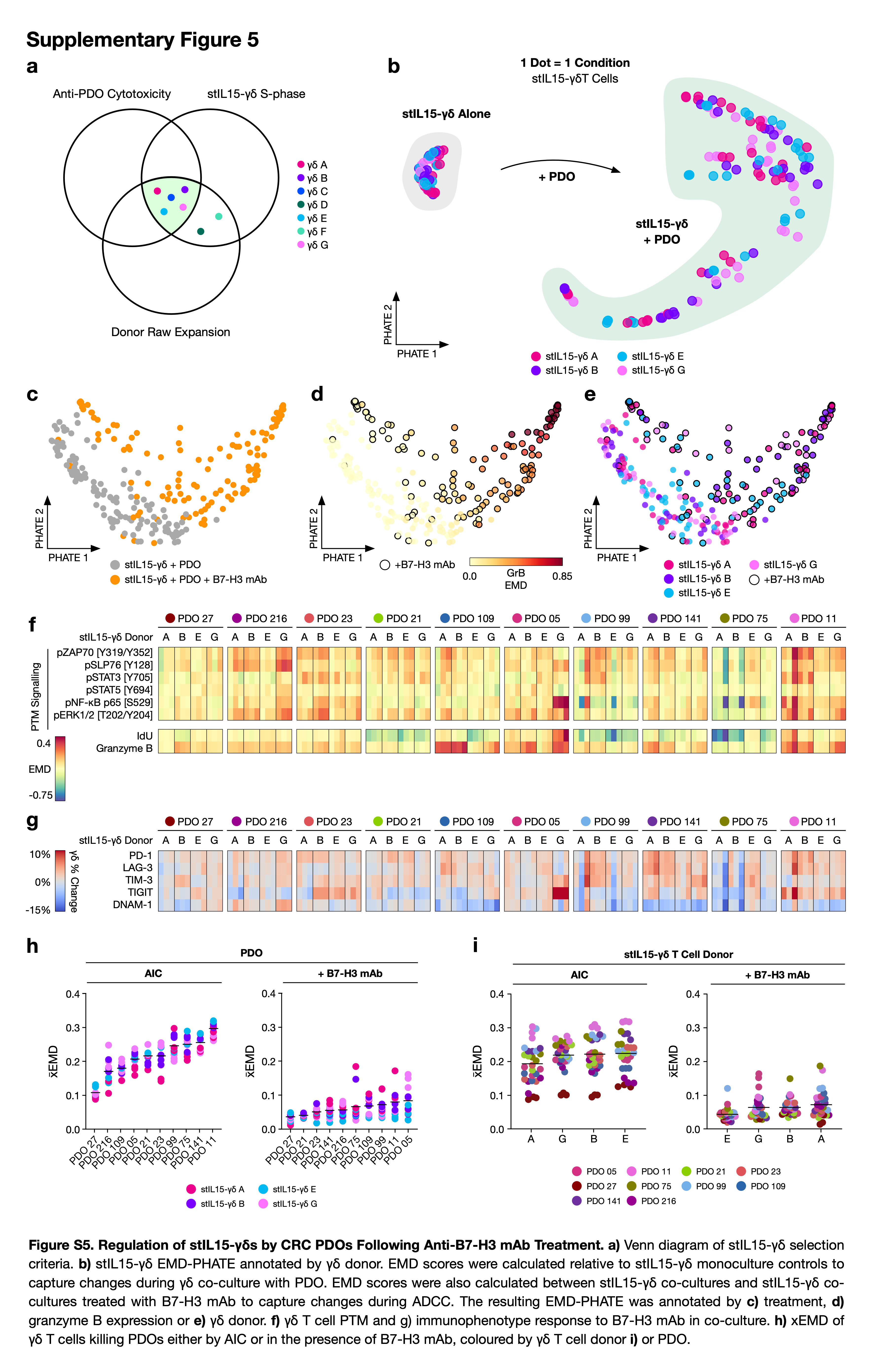

Supplement: Figure S5 — Regulation of stIL15-γδs signalling and immunophenotype by CRC PDOs with or without anti-B7-H3 mAb [file can-25-1890_figure_s5_suppsf5.png]

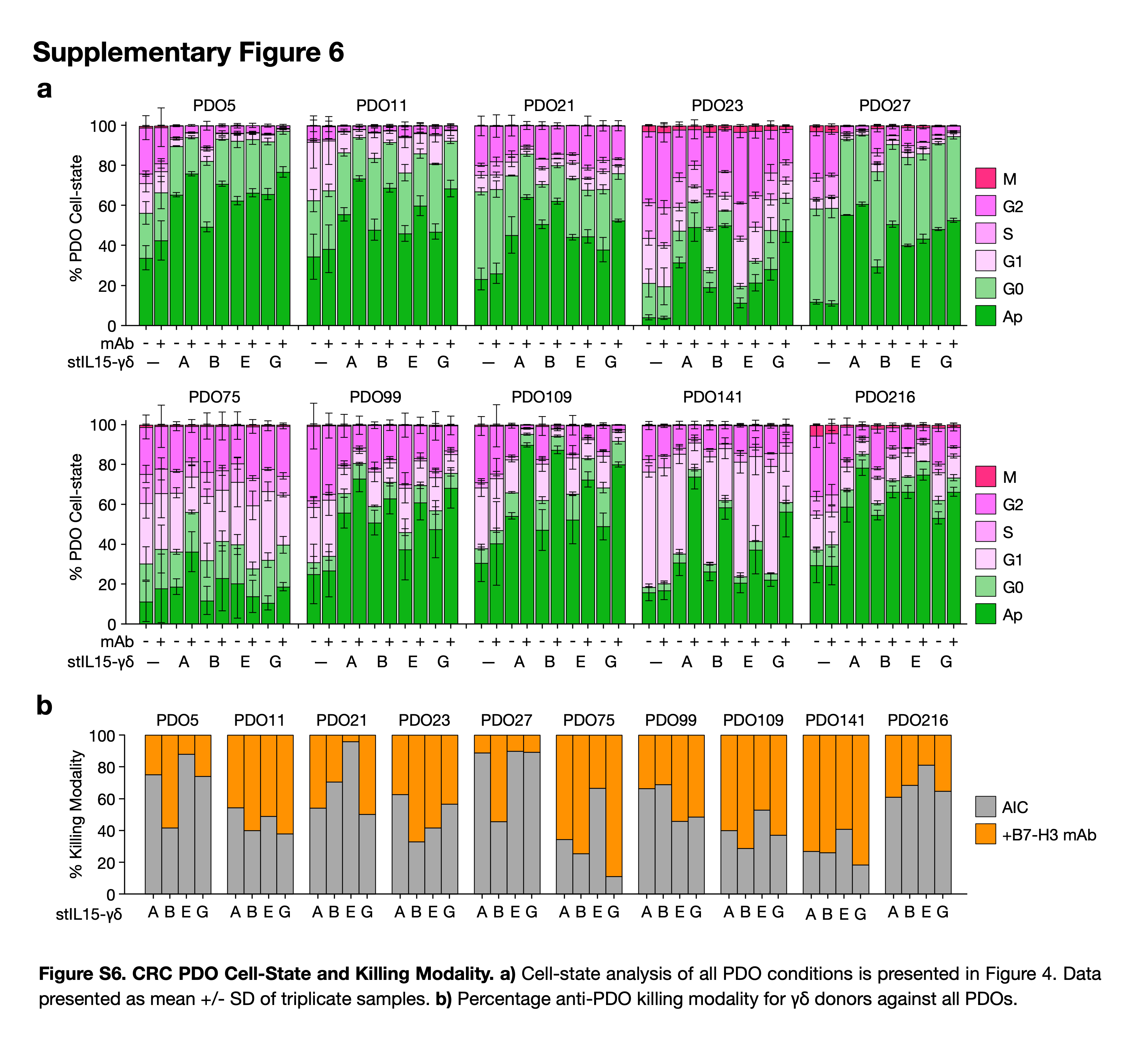

Supplement: Figure S6 — CRC PDO cell-state and γδT cell cytotoxic modality [file can-25-1890_figure_s6_suppsf6.png]

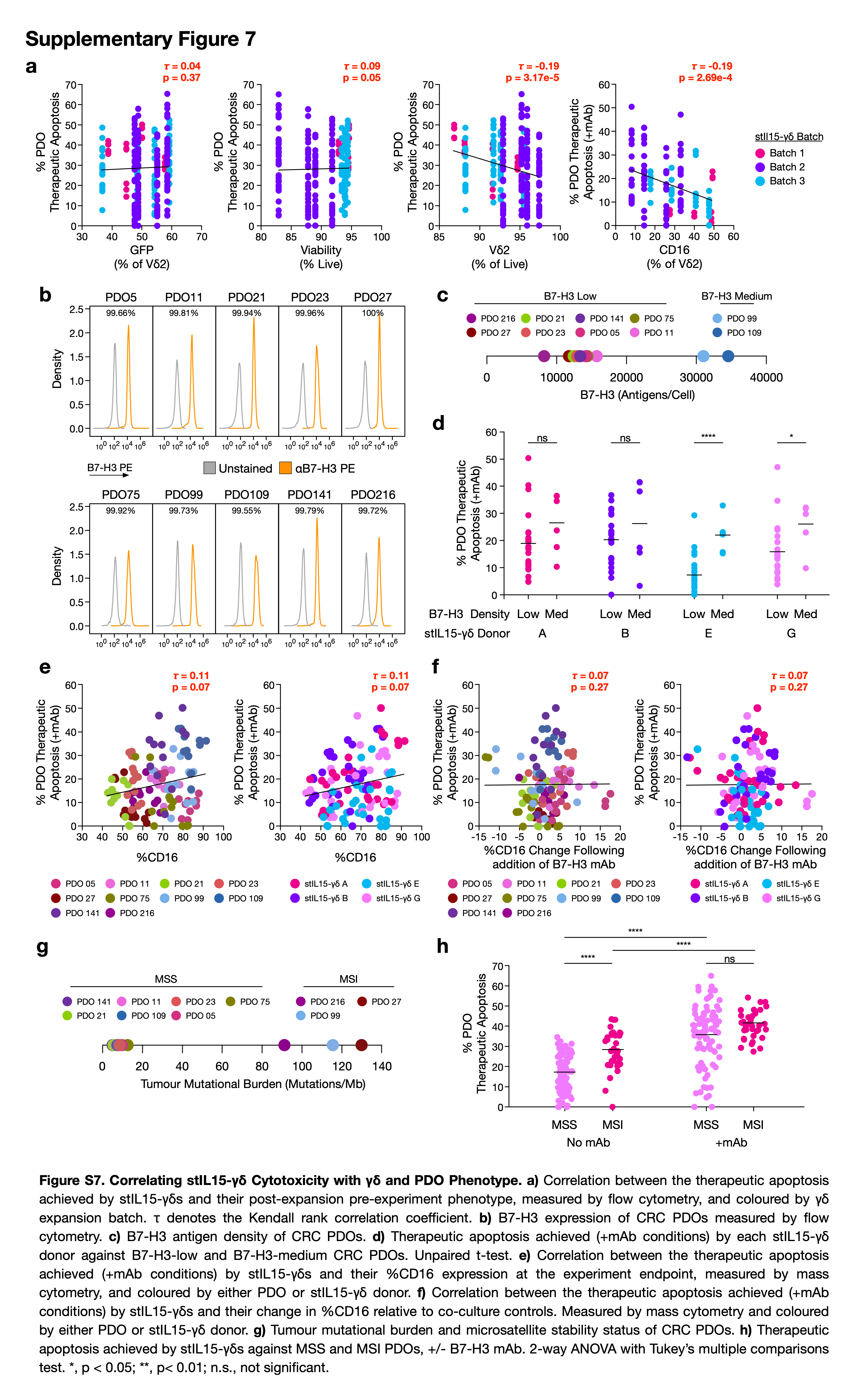

Supplement: Figure S7 — Relationship between stIL15-γδ cytotoxicity and γδ T cell and PDO phenotypes [file can-25-1890_figure_s7_suppsf7.png]

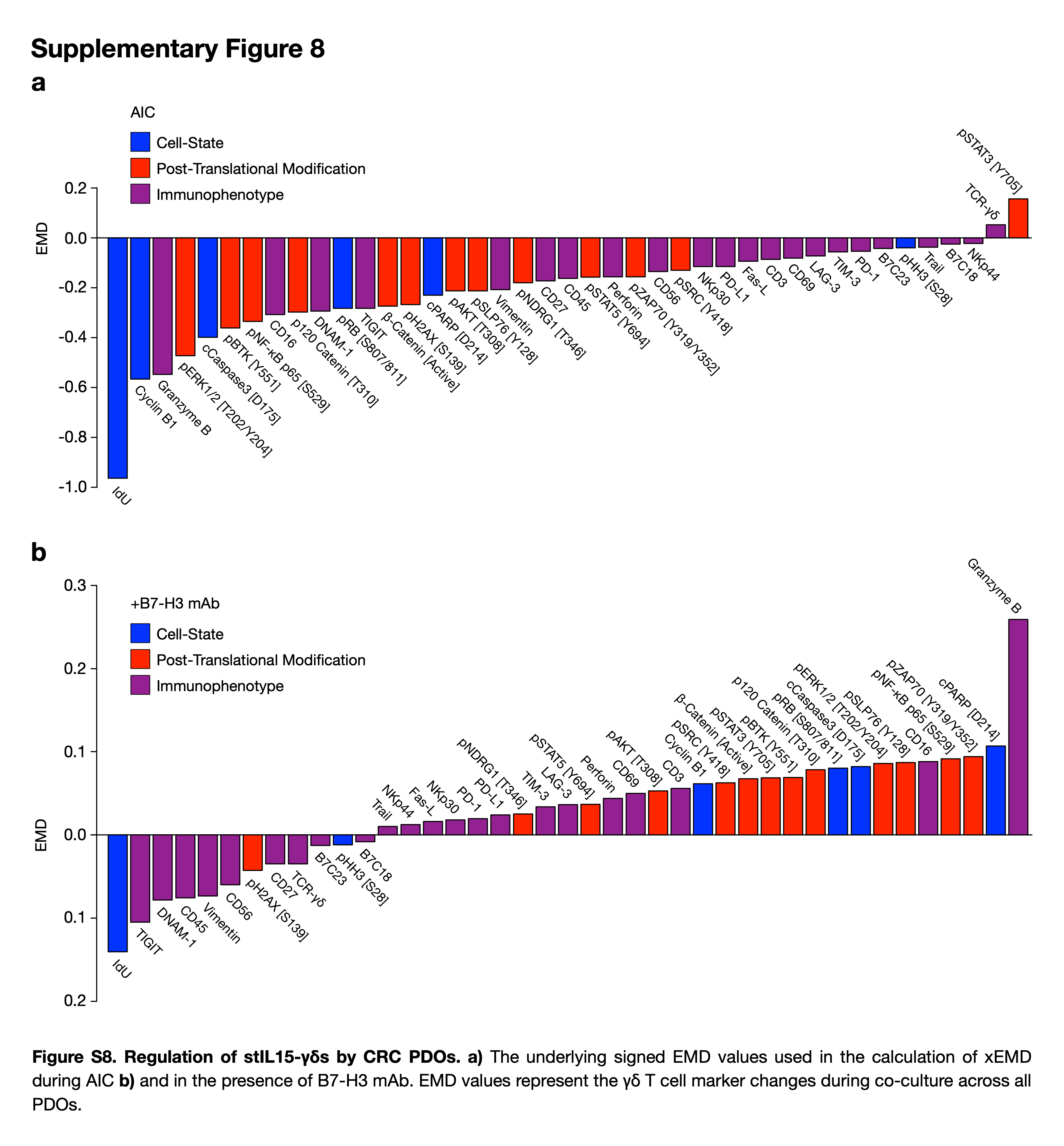

Supplement: Figure S8 — stIL15-γδ T cell regulation by CRC PDOs with or without anti-B7-H3 mAb [file can-25-1890_figure_s8_suppsf8.png]

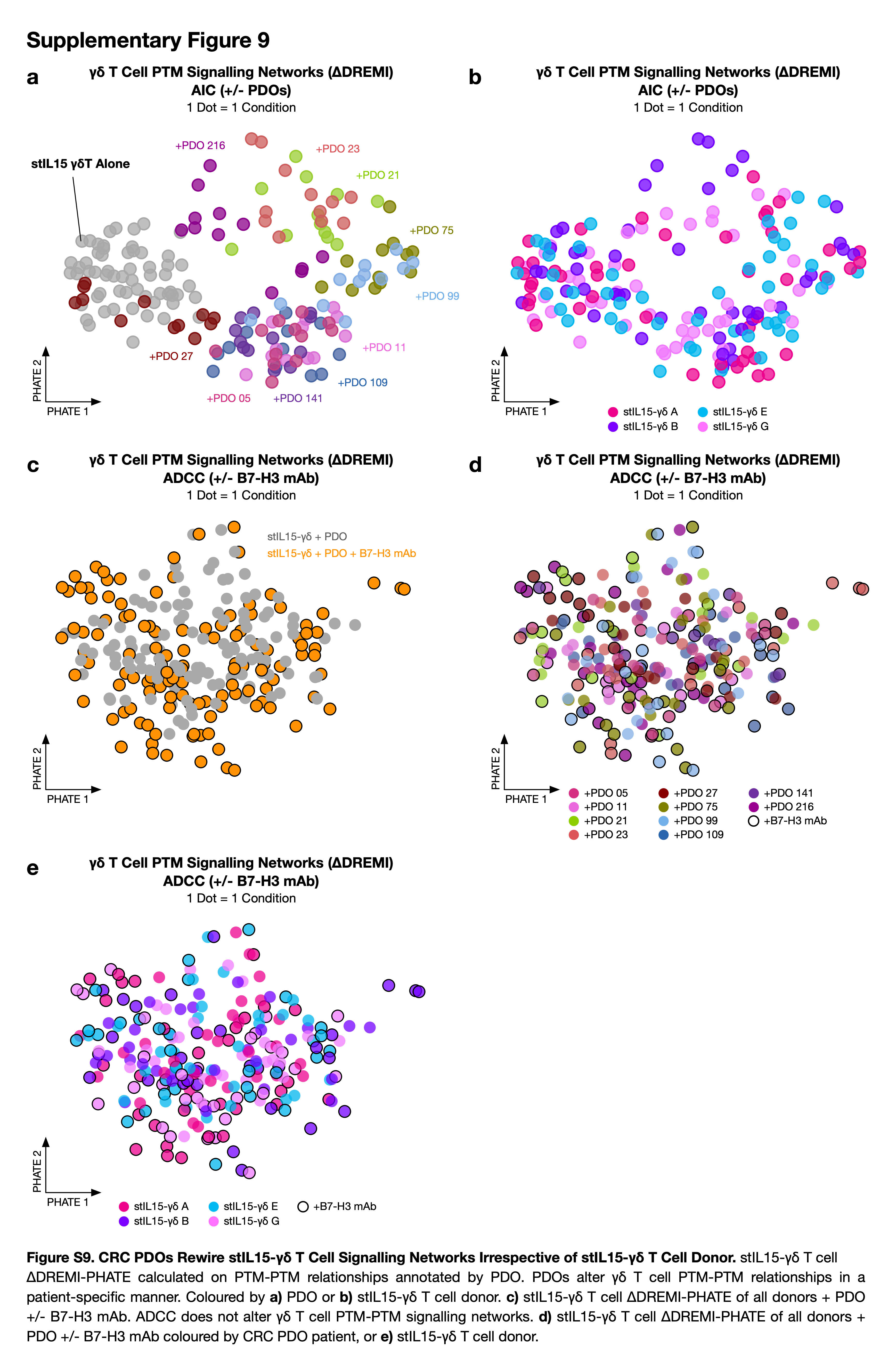

Supplement: Figure S9 — DREMI rewiring of stIL15-γδ T cell signalling networks by CRC PDOs across all stIL15-γδ T cell donors [file can-25-1890_figure_s9_suppsf9.png]
